# Determinants of accident and emergency attendances and emergency admissions in infants: birth cohort study

**DOI:** 10.1186/s12913-022-08319-1

**Published:** 2022-07-21

**Authors:** Selina Nath, Ania Zylbersztejn, Russell M. Viner, Mario Cortina-Borja, Kate Marie Lewis, Linda P. M. M. Wijlaars, Pia Hardelid

**Affiliations:** Population, Policy and Practice Research & Teaching Department, UCL Great Ormond Street Institute of Child Health, 30 Guilford Street, London, WC1N 1EH UK

**Keywords:** Emergency admissions, Emergency care, Infant health, Local authority, Variations, Hospital episode statistics

## Abstract

**Background:**

There is limited understanding of the drivers of increasing infant accident and emergency (A&E) attendances and emergency hospital admissions across England. We examine variations in use of emergency hospital services among infants by local areas in England and investigate the extent to which infant and socio-economic factors explain these variations.

**Methods:**

Birth cohort study using linked administrative Hospital Episode Statistics data in England. Singleton live births between 1-April-2012 and 31-March-2019 were followed up for 1 year; from 1-April-2013 (from the discharge date of their birth admission) until their first birthday, death or 31-March-2019.

Mixed effects negative binomial models were used to calculate incidence rate ratios for A&E attendances and emergency admissions and mixed effects logistic regression models estimated odds ratio of conversion (the proportion of infants subsequently admitted after attending A&E). Models were adjusted for individual-level factors and included a random effect for local authority (LA).

**Results:**

The cohort comprised 3,665,414 births in 150 English LAs. Rates of A&E attendances and emergency admissions were highest amongst: infants born < 32 weeks gestation; with presence of congenital anomaly; and to mothers < 20-years-old. Area-level deprivation was positively associated with A&E attendance rates, but not associated with conversion probability. A&E attendance rates were highest in the North East (916 per 1000 child-years, 95%CI: 911 to 921) and London (876 per 1000, 95%CI: 874 to 879), yet London had the lowest emergency admission rates (232 per 1000, 95%CI: 231 to 234) and conversion probability (25% vs 39% in South West). Adjusting for individual-level factors did not significantly affect variability in A&E attendance and emergency admission rates by local authority.

**Conclusions:**

Drivers of A&E attendances and emergency admissions include individual-level factors such being born premature, with congenital anomaly and from socio-economically disadvantaged young parent families. Support for such vulnerable infants and families should be provided alongside preventative health care in primary and community care settings. The impact of these services requires further investigation. Substantial geographical variations in rates were not explained by individual-level factors. This suggests more detailed understanding of local and underlying service-level factors would provide targets for further research on mechanisms and policy priority.

**Supplementary Information:**

The online version contains supplementary material available at 10.1186/s12913-022-08319-1.

## Background

Hospital contact rates among children peak during infancy (< 1 year old) [1–5], with the proportion of ‘inappropriate’ accident and emergency (A&E) attendances (attendances to an emergency department that did not lead to an admission or any treatment/intervention) also being highest among infants [6]. The most frequent reasons for infant admissions via A&E departments include bronchiolitis, upper respiratory viral, intestinal infections [2], gastroenteritis, jaundice and feeding difficulties [7]. Over the last 15 years, England has experienced substantial increases in both A&E attendances and subsequent emergency admissions for infants; from 280 per 1000 infants in 2004 to 389 per 1000 infants in 2018 [2, 3, 7–12]. The health system in England is a national, primary-care led free at the point of access system. It is divided into primary care, delivered in the community by general practitioners (GPs) and secondary or hospital-based care, accessed through presentation to A&E or by GP referral. Health policy is largely determined by central government [13]. Therefore, patients are not likely to avoid presenting to A&E because of worries about the cost of care. Several policy changes since 2000 may have contributed to the increasing trends in admissions amongst children, including targets to reduce patients waiting for > 4-hours implemented across all National Health Services (NHS) A&E departments in England by 2000–2003 (which may have led to increases in short-term hospital admissions, particularly to short-stay assessment units [14]), changes to GP contracts in 2004 leading to reduced out-of-hours services [10], and reductions in funding for postnatal health visiting services [15, 16].

There are marked regional variations in rates of A&E attendances and subsequent emergency hospital admissions for infants across England, particularly between London and elsewhere. In 2018, for example, there were 1,191 A&E attendances per 1000 infants in London (compared to 957 per 1000 infants in England overall) [17], but 261 emergency admissions per 1000 infants in London (compared to 389 per 1000 infants in England overall) [18]. Reasons for such large variations are likely complex and multifactorial, highlighting the importance of considering individual-level maternal and infant risk factors for emergency hospital care [19, 20]. Improving our understanding of the drivers of geographical variation in A&E attendances and subsequent emergency hospital admissions for infants is, therefore, a key public health issue [21] and would enable us to determine the extent to which rates are driven by different population needs or other service related factors. As local authorities (LAs) are responsible for improving the population health, public health services and service delivery at a local level, it is important to further investigate hospital contact rates by LAs in England.

The NHS Long Term Plan outlines policies intended to relieve the pressure on A&E departments by increasing both ‘out-of-hours’ and ‘out-of-hospital’ care starting from 2019/20 [22]. The NHS Outcomes Framework specified reducing emergency admissions for conditions that are manageable outside of hospital as an indicator and target for health improvement [23]. The Royal College of Paediatrics and Child Health (RCPCH) proposed specific strategies aimed to reduce children’s A&E attendances and emergency admissions including ‘GP hotlines’ enabling direct communication between primary care and paediatricians and ‘Hospital at Home’ services [14, 24, 25]. Understanding variations and drivers of infant hospital use would enable implementation and targeting of policies to support these strategies. Currently there are guidelines from the NHS and National Institute of Health and Care Excellence (NICE) which outline factors related to the child and family to consider when admitting a child, such as those for fever recommendations [26, 27]. However, there are likely variations in compliance with such guidelines, e.g. as shown in with bronchiolitis management [28].

The primary aim of this study was to examine the local variation in use of emergency hospital services among infants in England and to explore whether infant and family socio-economic factors explain this variation. The secondary aim was to examine whether infant and family socio-economic factors are associated with the probability of being admitted after having attended an A&E department (conversion probability).

## Methods

### Data sources

This is a population-based birth cohort study using Hospital Episode Statistics (HES), a hospital administrative database containing data from all National Health Service (NHS) hospitals in England. HES captures data on all A&E attendances (HES A&E), admitted patient care (HES APC) admissions and outpatient appointments in NHS-funded hospitals across England. HES data include a unique pseudonymised patient identifier (the HESID) which enables longitudinal linkage of children’s hospital contacts over time [29, 30]. We used a previously constructed nationally representative birth cohort generated using HES APC birth episodes linked to maternal delivery episodes, which includes all singleton children born in NHS hospitals to English resident mothers. The cohort includes 97% of all births in England; full details of the birth cohort derivation are described elsewhere [31, 32].

We extracted data on all A&E attendances and emergency admissions for infants in the birth cohort from HES A&E and HES APC respectively. We used the methods proposed by NHS Digital for linking A&E attendances and emergency admissions (described in online supplementary materials S1) [33, 34].

### Study population and follow-up

**Fig. 1:**
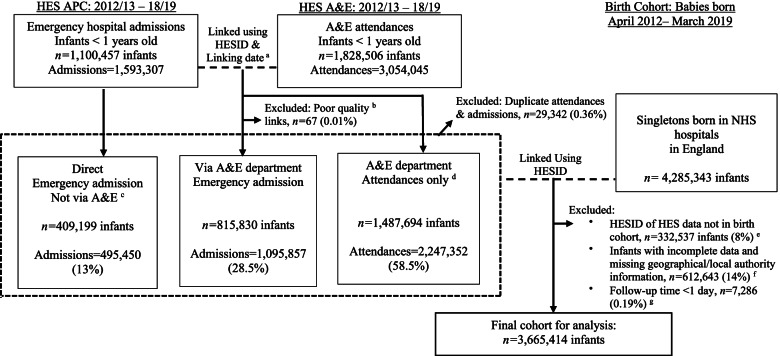
Flow chart of linkage between HES APC, HES A&E and birth cohort data. Notes: ^a^ Linking date was discharge date of A&E attendance (from HES A&E dataset) and date of hospital emergency admission (from HES APC dataset). ^b^ Quality control was conducted on linked A&E and APC records according to methodology guidelines from NHS digital. 90% were strong links, 10% good links and only 0.01% poor links (*n* = 67) which were removed as recommended by the guidelines. Emergency admissions via A&E department that did not link to an A&E attendance record showed 17% were coded as 21 (A&E department) and 83% as code 28 (other means). We prioritised information from APC dataset to indicate emergency admission via A&E department. ^c^ Direct emergency admissions were admission method codes 22 (GP,92%), 23 (bed bureau, 2%), & 24 (Consultant clinic, 6%) from HES APC dataset. These were removed prior to linkage and appended in afterwards. ^d^ All of the A&E attendances that were not linked to an APC emergency admission were assumed to be an attendance without an admission. ^e^ Of observations that did not link with UCL birth cohort, the majority were HESID’s from A&E data (63%). Whereas, 26% were HESID’s found in the A&E and APC linked data, and 11% from APC data only (direct admissions). 48% of the non-linking records were from 2012/13 and therefore may have been records from older infants that were not in the birth cohort (i.e. born before March 2012). This suggests that those not linked might be due to HESID data quality issues in the A&E dataset and HES records from older infants not in the birth cohort for the first year. ^f^ 701,680 infants had missing data on any risk factor (16%) and were excluded from the analysis sample; missing values were slightly more frequent in later study years (OR: 1.025, 95%CI: 1.024–1.046). ^g^ As the first year 2012/13 did not have full follow-up data from infant born in previous year, we dropped attendances and admissions in 2012/13. The rest of the financial years consisted of full follow-up

We included all children in the cohort born between 1^st^ April 2012 and 31^st^ March 2019. Infants were followed up from 1^st^ April 2013 (start of follow-up) or discharge date of their birth admission (whichever occurred later), to date of first birthday, date of death, estimated date of migration or end of follow-up on 31^st^ March 2019 (whichever came first). The study period ensured that all study years included follow-up for infants aged up to 12 months and excluded any time spent in hospital. Infants who moved out of England were identified by the presence of a non-English postcode for any A&E attendance or emergency admission during the study period. The date of emigration was set at mid-point between the infant’s start of follow-up date and the date at which the non-resident attendance or admission was recorded. Infants born < 24 weeks gestation and multiple births were excluded. Figure 1 shows the flow-chart of data linkage and final sample of cohort infants used in the analysis.

### Outcomes

The study’s primary outcomes were rates of A&E attendances and rates of emergency admissions in infants. The secondary outcome was the conversion probability, i.e. the proportion of children admitted to hospital after attending A&E.

#### A&E attendances

A&E attendances were identified using the HES A&E dataset and defined as an unplanned attendance to a 24-hour consultant-led A&E department, consultant-led mono specialty A&E department (e.g. ophthalmology or dental A&E departments), and other types of A&E departments and minor injury units. Follow-up A&E attendances, attendances at NHS walk-in centres, and A&E attendances in private hospitals were excluded. For infants with more than one A&E attendance in one day, only the last attendance was included to avoid double counting and allow linkage with a potential subsequent emergency admission.

#### Emergency admissions

The HES APC dataset was used to identify emergency admissions. We identified two distinct pathways to an emergency admission within the APC dataset using the variable ‘admission method’. The first was via a hospital A&E attendance (indirect), where parents/carers would take infants to an A&E department in a hospital and the infant was then admitted as an emergency from the A&E department (also called conversion). The second was a direct hospital admission (circumventing an A&E attendance, often recommended by a general practitioner; GP). We included all emergency admissions (irrespective of admission pathway) for analysis. Admissions were classified as an emergency if the first episode of care within a multi-episode admission was recorded as an emergency episode.

### Infant and socio-economic risk factors

Infant birth characteristics included infant’s sex, gestational age at birth defined as severe prematurity (< 32 weeks), moderate prematurity (32 – 33 weeks), near term (34 – 36 weeks), and term (≥ 37 weeks) [35], and congenital anomalies, identified using an International Classification of Diseases version 10 (ICD10) code list [32, 36]. Children were classified as having a congenital anomaly if a relevant code was recorded at birth, during any hospital admission before 2 years of age, or on a death certificate as any cause of death. Maternal age was used as a family-level indicator of socio-economic status and categorised as under 20, 20—29, 30—39 and 40 + years at the time of delivery [37]. Quintiles of the Index of Multiple Deprivation score (IMD, 2010 version) were used as a further indicator of family socio-economic status. The IMD is a composite measure of multiple deprivation at Lower Super Output Area level (covering 1500 people on average) across seven domains [38, 39]. HES financial years (running April to March in the UK) were used to ensure comparison of annual rates published by Public Health England (PHE) [17, 18, 40].

We used information about infant’s local authority (LA) of residence at birth to examine variations in A&E attendances and emergency admissions across English local areas. There were 152 LAs (upper tier) across England during the study period. Due to small population sizes (< 1000 births across the study period), we combined the City of London with Hackney, and the Isles of Scilly with Cornwall. This resulted in 150 LAs included in the analyses. LAs were grouped into region of residence (North East, North West, Yorkshire and Humber, East Midlands, West Midlands, East of England, London, South East, and South West) to describe overall patterns of A&E attendances and emergency admissions at a regional level in England.

### Statistical methods

We described distribution of region, infant and maternal risk factors for children in the cohort (overall and in complete-case cohort excluding children with missing data). Our main analyses were restricted to a complete-case cohort. Rates of A&E attendances and emergency admissions were calculated per 1000 child-years, overall and stratified according to all risk factors [41].

We fitted mixed-effects negative binomial regression models to examine the association between infant and maternal risk factors and rates of A&E attendances and admissions, and derive incidence rate ratios (IRRs) [42]. The outcome variable was counts of A&E attendances or emergency admissions; separate models were fitted for each outcome. Year and month of birth (categorised as standard calendar quarters), infant’s sex and gestational age, presence of congenital anomaly, maternal age, and quintiles of IMD were included as exposure variables, and person-time at risk as the offset. Risk factors included in the models were selected a priori, based on previous literature [5, 12, 43–46]. Year and month of birth were included in the first model (model 1), then infant-related variables (infant sex, gestational age, presence of congenital anomaly) were added (model 2), and finally, maternal age, and quintiles of IMD were included (model 3). Heterogeneity due to unobserved variables within LAs was accounted for with a random effect term in the intercept of the models. Variance Partition Coefficients (VPC) defined as:

1 $$\frac{\sigma_u^2}{\sigma_u^2+\sigma_e^2}$$

that is, as the ratio of the residual variance due to between-LA random effects (${\sigma }_{u}^{2})$ and the total residual variance (${\sigma }_{e}^{2})$. Thus, VPC can be interpreted as the proportion of the total residual variance attributable to the random effects [42, 47]. In other words, the proportion of variation that is beyond that explained by the fixed predictors that is due to between‐LA variation.

We assessed goodness-of-fit for all models by changes in the Bayesian information criterion (BIC) [48], with smaller values indicating better model fit. Normal probability plots of deviance residuals were used to determine deviations outside of the normal expected range, as only 5% of deviance residuals should lie outside ± 1.96. The assumption of normality for the models’ random effects was assessed with probability plots of the individual random effects predictions.

To visually examine LA level variation in rates of A&E attendances and emergency admissions, we used crude and adjusted rates to construct funnel plots with multiplicative over-dispersion adjusted control limits. Expected events were obtained from the final model’s predictions using both the fixed effects estimates and the LA random effect term (model 3). Adjusted rates were then calculated as the observed number of events divided by the expected number of events obtained using predictions from the final model for each outcome, multiplied by the overall crude rate for England [49]. To further explore the contribution of infant and socio-economic factors within the model, we compared LA effect predictions from the final adjusted mixed-effects models (model 3) against those from model 1 (i.e., the null model). Maps were generated to show variations of adjusted rates (per 1000 child-years) of A&E attendances, emergency admissions and conversation probabilities by local authorities across England.

#### Models for conversion probabilities

Mixed-effects logistic regression models were fitted to determine the association between individual-level infant and maternal characteristics, and the probability of being admitted to hospital given that the infant had attended A&E (the conversion probability). These models were parametrised in terms of odds ratios. We included the same covariates and used the same model selection strategy as for the negative binomial regression models described above. LA was included as a random effect in the models’ intercept. For this analysis, we selected at random one A&E attendance per infant for those with multiple A&E attendances during infancy. VPC was calculated using:

2 $$\frac{\sigma_u^2}{\sigma_u^2+{\displaystyle\frac{\pi^2}3}}$$

where ${\sigma }_{u}^{2}$ is the variance of the within LA random effects (level 2), and the level 1 variance for a logistic regression model is obtained as the variance of the standard logistic distribution, $\frac{{\pi }^{2}}{3}$ [50].

#### Sensitivity analyses

Logistic regression models were used to examine factors that were associated with missingness (outcome: complete data vs any missing data) showing that lower gestational age was significantly associated with increased odds of missing information. To investigate the potential influence of missing data on the results, we ran all final models including an additional category of missing to the infant gestational age category. All statistical analysis were conducted in STATA v16 [51].

## Results

### Study population

The distribution of infant, maternal and geographical variables with complete (*n* = 3,672,700) and missing data (*n* = 612,643) are shown in online supplementary table S2. Infant’s gestation at birth had the most missing data, compared to other variables (12.5% missing data vs < 3% for other variables) and missing data on any variable was more common for preterm born (< 37 weeks) infants (OR 2.03, 95%CI 1.92–2.16). Figure 1 shows flow-chart of the final study cohort. Of all singleton births in our complete dataset, 0.19% (*n* = 7,286) had follow-up < 1 day and were excluded from further analyses. The final study cohort included 3,665,414 singleton infants from 150 LAs, with mean follow-up time per infant of 342 days (median = 365 days). There were 2,241,892 A&E attendances and 1,051,619 emergency admissions during the study period; 716,838 (68%) emergency admissions were via A&E and 334,781 (32%) were admissions directly to a ward. Of the direct admissions, 307,498 (92%) were from GPs.

**Fig. 2:**
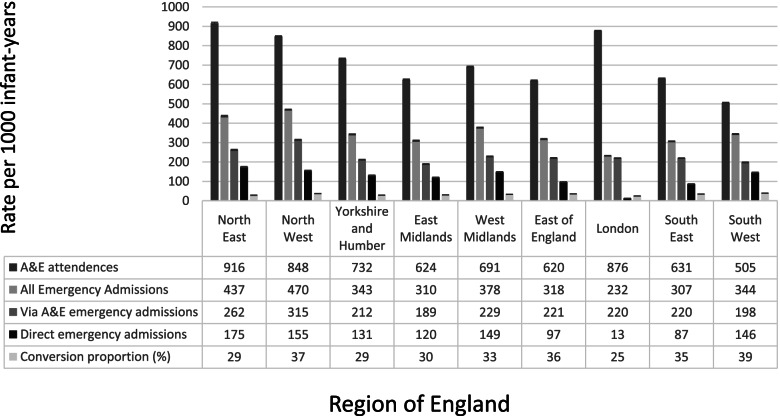
A&E attendances and emergency admissions for infants < 1 years old in England by Region of residence

Table 1 shows the distribution of characteristics in the study cohort included in the analysis. The overall A&E attendance rate was 720 per 1000 child-years (95%CI: 719–721) and the emergency hospital admission rate 338 per 1000 child-years (95%CI: 337–338). The annual rates by financial years broadly followed published rates (see Table 1) [17, 18]. We identified substantial regional variations in A&E attendance and hospital admission rates. A&E attendance rates were highest in the North East (916 per 1000 child-years, 95%CI: 911–921) and London (876 per 1000 child-years, 95%CI: 874–879), yet London had the lowest emergency admission rates (232 per 1000 child-years, 95%CI: 231–234) and lowest conversion proportion (25%) (see Fig. 2). Infants who were male, born at < 32 weeks’ gestation or with congenital anomalies, from the most deprived SES quintile or with young mothers had higher A&E attendance and admission rates.

**Table 1:** Cohort sociodemographic and rates (95%CI) of A&E attendance and emergency admissions

	All infants		A&E attendances of infants		Emergency admissions of infants	
	*n*	%	Count (%)	Rate per 1000 infant-years(95%CI)	Count (%)	Rate per 1000 infant-years(95%CI)
Total	3,665,414	100	2,241,892 (100)	720.1 (719.2 to 721.1)	1,051,619 (100)	337.8 (337.1 to 338.4)
**Year**	Year of birth		Year of A&E attendance ^a^		Year of emergency admission ^b^	
2012/13	561,558	15				
2013/14	532,303	15	343,220 (15.3)	634.1 (632 to 636.2)	168,690 (16.0)	311.7 (310.2 to 313.2)
2014/15	523,287	14	333,812 (14.9)	639.4 (637.2 to 641.6)	166,616 (15.8)	319.1 (317.6 to 320.7)
2015/16	526,828	14	367,478 (16.4)	706.6 (704.3 to 708.9)	174,781 (16.6)	336.1 (334.5 to 337.6)
2016/17	517,687	14	394,000 (17.6)	762.0 (759.6 to 764.4)	182,495 (17.4)	352.9 (351.3 to 354.6)
2017/18	522,047	14	395,102 (17.6)	768.0 (765.6 to 770.4)	176,731 (16.8)	343.5 (341.9 to 345.1)
2018/19	481,704	13	408,280 (18.2)	819.3 (816.8 to 821.8)	182,306 (17.3)	365.8 (364.2 to 367.5)
**Month of birth**						
January	301,035	8	183,634 (8.2)	713.5 (710.3 to 716.8)	84,619 (8.0)	328.8 (326.6 to 331)
February	270,279	7	164,126 (7.3)	707.3 (703.9 to 710.7)	75,879 (7.2)	327.0 (324.7 to 329.3)
March	286,214	8	174,659 (7.8)	704.4 (701.1 to 707.7)	81,002 (7.7)	326.7 (324.4 to 328.9)
April	299,542	8	180,024 (8.0)	715.5 (712.2 to 718.8)	83,205 (7.9)	330.7 (328.4 to 332.9)
May	316,786	9	191,011 (8.5)	718.6 (715.4 to 721.9)	88,419 (8.4)	332.7 (330.5 to 334.9)
June	308,684	8	188,949 (8.4)	725.7 (722.4 to 729)	87,813 (8.4)	337.3 (335 to 339.5)
July	323,267	9	198,965 (8.9)	726.8 (723.6 to 730)	93,189 (8.9)	340.4 (338.2 to 342.6)
August	318,448	9	195,863 (8.7)	727.5 (724.3 to 730.7)	91,883 (8.7)	341.3 (339.1 to 343.5)
September	321,338	9	197,154 (8.8)	723.6 (720.4 to 726.8)	95,187 (9.1)	349.4 (347.1 to 351.6)
October	319,677	9	197,791 (8.8)	728.5 (725.3 to 731.7)	97,303 (9.3)	358.4 (356.1 to 360.6)
November	301,002	8	186,145 (8.3)	727.1 (723.8 to 730.4)	88,768 (8.4)	346.7 (344.5 to 349)
December	299,142	8	183,571 (8.2)	719.5 (716.2 to 722.8)	84,352 (8.0)	330.6 (328.4 to 332.9)
**Sex**						
Male	1,881,844	51	1,250,909 (55.8)	783.2 (781.8 to 784.6)	601,658 (57.2)	376.7 (375.8 to 377.7)
Female	1,783,570	49	990,983 (44.2)	653.6 (652.4 to 654.9)	449,961 (42.8)	296.8 (295.9 to 297.7)
**Gestation at birth**						
37 + Term	3,457,537	94	2,039,640 (91.0)	693.2 (692.3 to 694.2)	929,728 (88.4)	316.0 (315.4 to 316.6)
34–36:Near Term	26,286	1	36,590 (1.6)	1926.3 (1906.6 to 1946.1)	25,803 (2.5)	1358.4 (1341.9 to 1375.1)
32–33:Moderate prematurity	23,959	1	23,818 (1.1)	1228.2 (1212.7 to 1243.9)	14,902 (1.4)	768.4 (756.2 to 780.9)
< 31:Severe &extreme prematurity	157,632	4	141,844 (6.3)	1068.6 (1063.1 to 1074.2)	81,186 (7.7)	611.6 (607.4 to 615.9)
**Congenital anomaly**						
No	3,550,426	97	2,076,303 (92.6)	687.6 (686.7 to 688.6)	933,323 (88.8)	309.1 (308.5 to 309.7)
Yes	114,988	3	165,589 (7.4)	1766.5 (1758 to 1775)	118,296 (11.2)	1262.0 (1254.8 to 1269.2)
**IMD Quintile** ^c^						
Q1: Most deprived	1,017,935	28	758,620 (33.8)	878.8 (876.8 to 880.8)	320,180 (30.4)	370.9 (369.6 to 372.2)
Q2	817,545	22	521,845 (23.3)	752.0 (750 to 754)	234,770 (22.3)	338.3 (337 to 339.7)
Q3	682,265	19	382,020 (17.0)	658.6 (656.5 to 660.7)	189,805 (18.0)	327.2 (325.8 to 328.7)
Q4	592,180	16	309,765 (13.8)	615.3 (613.1 to 617.4)	162,995 (15.5)	323.7 (322.2 to 325.3)
Q5.Least deprived	555,490	15	269,640 (12.0)	570.6 (568.4 to 572.7)	143,870 (13.7)	304.4 (302.9 to 306)
**Region** ^c^						
North East	169,965	5	132,465 (5.9)	916.0 (911.1 to 920.9)	63,135 (6.0)	436.6 (433.2 to 440)
North West	496,105	14	356,515 (15.9)	847.7 (844.9 to 850.5)	197,670 (18.8)	470.0 (467.9 to 472.1)
Yorkshire and Humber	363,085	10	223,945 (10.0)	732.3 (729.2 to 735.3)	104,740 (10.0)	342.5 (340.4 to 344.6)
East Midlands	298,845	8	156,680 (7.0)	624.3 (621.2 to 627.4)	77,740 (7.4)	309.8 (307.6 to 312)
West Midlands	406,340	11	239,290 (10.7)	691.5 (688.7 to 694.3)	130,660 (12.4)	377.6 (375.5 to 379.6)
East of England	376,090	10	197,060 (8.8)	619.5 (616.8 to 622.3)	101,150 (9.6)	318.0 (316.1 to 320)
London	655,480	18	485,830 (21.7)	876.2 (873.8 to 878.7)	128,735 (12.2)	232.2 (230.9 to 233.5)
South East	554,670	15	301,160 (13.4)	630.4 (628.2 to 632.7)	146,355 (13.9)	306.4 (304.8 to 308)
South West	344,830	9	148,940 (6.6)	504.9 (502.3 to 507.4)	101,430 (9.6)	343.8 (341.7 to 345.9)
**Maternal Age**						
Under 20	133,057	4	126,233 (5.6)	1133.1 (1126.8 to 1139.3)	53,553 (5.1)	480.7 (476.6 to 484.8)
20—29	1,627,985	44	1,113,349 (49.7)	805.4 (803.9 to 806.9)	520,738 (49.5)	376.7 (375.7 to 377.7)
30—39	1,759,622	48	927,821 (41.4)	619.9 (618.6 to 621.2)	442,373 (42.1)	295.6 (294.7 to 296.4)
40 +	144,750	4	74,489 (3.3)	606.5 (602.2 to 610.9)	34,955 (3.3)	284.6 (281.7 to 287.6)

^a^ Public Health England fingertips reported rates for annual A&E attendances in infants < 1 years old were 688.3 (2013/14), 719.6 (2014/15), 798.6 (2015/16), 859.9 (2016/17), 885.1 (2017/18) and 957.4 (2018/19) per 1000 population [15]. Although our rates were lower potentially due to additional data cleaning, linkage to birth cohort of singleton infants born in the UK and exclusion of those with missing data, the pattern of rates by year were comparable

^b^ Public Health England fingertips reported rates for annual emergency admissions in infants < 1 years old were 326.3 (2013/14), 338.1 (2014/15), 357.7 (2015/16), 369.0 (2016/17), 365.3 (2017/18) and 388.6 (2018/19) per 1000 population [16]. Although our rates were lower potentially due to additional data cleaning, linkage to birth cohort of singleton infants born in the UK and exclusion of those with missing data, the pattern of rates by year were comparable

^c^ NHS Digital Hospital Episode Statistics (HES) disclosure states all HES sub-national data is subject to suppression and rounded to the nearest 5. Therefore, counts for IMD Quintile and Region were rounded accordingly [30]

### A&E attendances

**Fig. 3:**
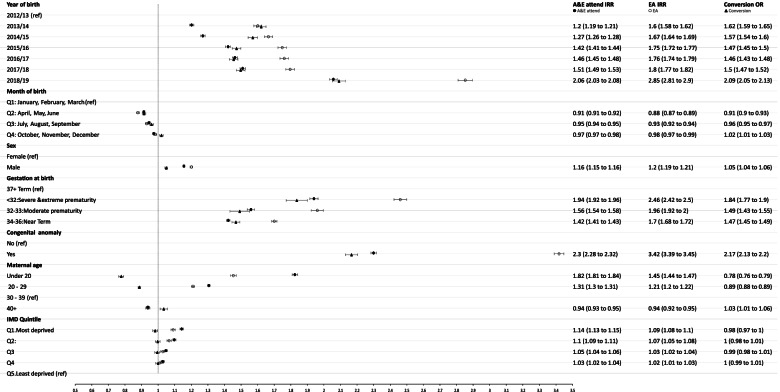
Adjusted IRRs for A&E attendences, Emergency Admissions (EA) and ORs for conversion (including 95% CI). Acronyms: IRR – Incidence Rate Ratio; OR – Odds Ratio; CI – Confidence Interval

A&E attendance rates were more than twice as high for infants who were born with congenital anomalies (IRR: 2.30, 95%CI: 2.28 to 2.32; Fig. 3) or at < 32 weeks (IRR: 1.94, 95%CI: 1.92 to 1.96) compared to those without congenital anomalies or born at full-term. Infants born to mothers delivering aged ≥ 40 years had lower rates of A&E attendances (IRR: 0.94, 95%CI: 0.93 to 0.95) and infants born to mothers aged under 20-years-old had 82% higher attendance rate than infants whose mothers were aged between 30 to 39 years (IRR: 1.82, 95%CI: 1.81 to 1.84). Infants from the most deprived SES quintile had 14% higher rates compared to those from the least deprived (IRR: 1.14, 95%CI: 1.13 to 1.15). Model 3 provided the best fit to A&E attendance rates (for full model outputs and model fit statistics see online supplementary materials table S3 and figure S7A).

**Fig. 4:**
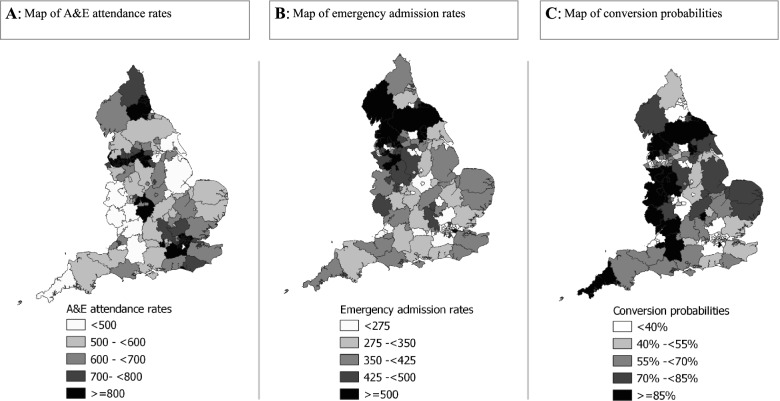
Maps showing adjusted rates (per 1000 child-years) of A&E attendences (**A**), emergency admissions (**B**) and converstion probabilities (**C**) by local authorities in England between finacial years 2013/14 – 2018/19

**Fig. 5:**
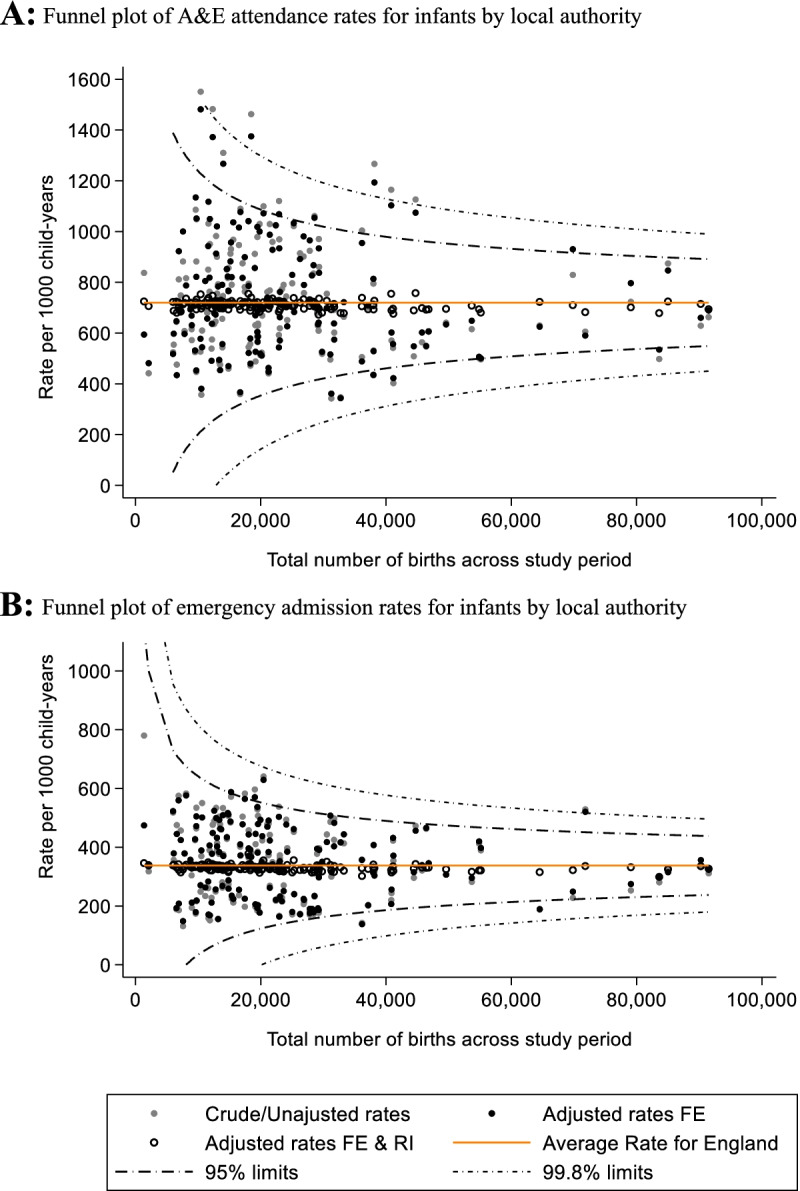
Funnel plots of unadjusted and adjusted A&E attendance (**A**) and emergency admission (**B**) rates by local authorities in England between financial years 2013/14 – 2018/19. **A**: Funnel plot of A&E attendance rates for infants by local authority. **B**: Funnel plot of emergency admission rates for infants by local authority. FE – Fixed effects, RI – Random Intercept

We identified substantial variation in adjusted A&E attendances rates by LA across England (Fig. 4A). Funnel plots of crude and adjusted A&E attendance rates in England showed that allowing for infant and socio-economic factors did not significantly change variations by LA (Fig. 5A). 17 (11%) LAs whose unadjusted crude rates fell outside the 95% control limits (vs 5% expected outside of the 95% control limits), similar to the 15 (10%) adjusted rates from LAs outside these limits. The VPC for adjusted model was 0.12.

Comparing predicted LA effect values from the final adjusted mixed effect models and the null model, showed a high correlation (online supplementary Figure S8A), confirming that infant and socio-economic factors explained little of the overall variations in A&E attendance rates (online supplementary Figure S9A for Normal probability plots of random effects predictions).

### Emergency admissions

Emergency admissions were more than three times higher in infants with congenital anomalies (IRR: 3.42, 95% CI: 3.39 to 3.45) and twice as high in infants born severely premature (IRR: 2.46, 95%CI: 2.42 to 2.50), compared to those born without congenital anomalies or full-term (Fig. 3). Emergency admission rates were 45% higher for infants whose mothers were < 20 years old, compared to infants whose mothers were 30 – 39 years-old (IRR: 1.45, 95%CI: 1.44 to 1.47). Model 3 had the best values of all goodness-of-fit statistics (for full model outputs and model fit statistics see online supplementary materials table S4 and figure S7B).

Allowing for infant and socio-economic factors did not substantially reduce the variation by LA (Figs. 4B and 5B). For the unadjusted crude rates only 6 (4%) LAs fell outside the 95% control limits, and 4 (3%) rates from the adjusted model, similar to the expected 5% normal variation outside of the 95% control limits. The VPC of the fully adjusted model was 0.12.

Predicted LA effect values from final adjusted mixed effect models and the null model, showed a high correlation (online supplementary Figure S8B). See online supplementary Figure S9B for normal probability plots of random effects predictions.

### Conversion probabilities

Of all infants in the cohort that had an A&E attendance during the study period, the overall conversion probability was 32% (716,838/2,241,892) and there were substantial variation across LAs in England (Fig. 4C). Compared to infants born in financial year 2012/13, infants born in subsequent financial years had increased odds of being admitted via A&E 2018/19 (OR: 2.09, 95%CI: 2.05 to 2.13) (see Fig. 3, online supplementary table S5 for all models and goodness-of-fit statistics, figure S10 and S11). ORs showed similar direction of trends as IRR for emergency admission although IMD quintile was not associated with conversion probabilities. The VPC of the fully adjusted model was 0.06.

### Sensitivity analysis

Including missing data category to gestational birth for all final adjusted models did not change the main findings substantially (supplementary table S6).

## Discussion

### Key results

Rates of emergency department use were highest amongst infants born at < 32 weeks gestation, with one or more congenital anomalies and to mothers < 20-years-old. Higher area-level deprivation was associated with higher A&E attendance rates and emergency admission rates, but not associated with conversion probability (A&E attendances resulting in admission). A&E attendance rates were highest in the North East and London, although London had the lowest emergency admission rates and conversion probabilities. We identified substantial variations in A&E attendance and emergency admission rates respectively by LA, that could not be explained by infant or socio-economic characteristics included in our models.

### Strengths and limitations

This was a large study including over 3 million infants, allowing us to account for multiple infant and socio-economic risk factors for A&E attendances and admissions, including some which are uncommon (eg severe prematurity). The strengths of our study include the use of a nationally validated birth cohort generated using administrative HES data covering 97% of births in England, minimising selection bias, extending beyond only those presenting at A&E, and ensuring inclusion of vulnerable groups (eg children with congenital anomalies). Full details of the birth cohort derivation are described elsewhere [31, 32]. Further linkage to HES A&E and APC records (99% high standard of quality linkage, 85% as strong linkage, 15% as good linkage) enabled us to follow-up infants during the first year of their life and include infant and socio-economic variables in the statistical models. However, 8% of A&E-APC records did not link to a birth cohort record, of which 89% were from the A&E dataset. Some of these records could have been from non-resident infants born outside of England (either on holiday or moved to England after birth), multiple births, and some may have been from infants whose mothers were asylum seekers/refugees without NHS numbers. The A&E dataset has been subject to data quality issues which likely also contribute to non-links [2]. Partly to address these data quality issues, NHS Digital have recently developed the new Emergency Care Data Set (ECDS) to replace the HES A&E dataset; a full evaluation of the ECDS for child health research has yet to be carried out [52]. Currently the ECDS data does not include a person-identifier, which prevents linkage of records longitudinally or to a birth cohort.

14% of births in the cohort were excluded as we used a complete-case analysis, which was mainly due to high proportion of missing data on infant gestational age. We therefore conducted sensitivity analysis including the missing gestational age to investigate any potential bias. The results were broadly similar, suggesting that our findings and conclusions are unlikely to be biased by missing data. Our birth cohort excluded multiple births to avoid increasing risks of false matches for multiple births [31, 53]. Due to limited data and quality issues on presentation and diagnoses in the HES A&E dataset, we did not investigate clinical condition severity or create definitions for appropriateness of attendances and admissions.

### Comparison with other studies and implications for policy

We found higher rates of A&E attendances, emergency admissions and conversion probabilities amongst infants born severely premature, with congenital anomalies and young mothers (< 20-years old). Similar findings on A&E attendances and emergency admissions have been reported in other studies conducted in England [5, 12, 45, 54]. It has been shown that despite substantial advances in the health care leading to in higher survival rates for preterm infants [55], these infants still remain at a higher risk of morbidity, including for acute infections, compared to infants born at full term [54, 56–59], which likely partly explains higher rates of A&E attendances [5] and conversion probabilities. Alternatively, our findings may reflect lower admission thresholds for preterm infants or those with comorbidities [60, 61]. Our study adds to the literature by investigating conversion from A&E to hospital admissions and demonstrates that the local area variation in A&E attendances and admission rates cannot be explained by infant or socio-economic factors.

Infants from the most deprived IMD quintile had 14% higher rates of A&E attendances compared to those from the least deprived. But of those that had an attendance, SES quintile was not associated with conversion probability. As conversion probabilities do not vary significantly between IMD quintile, it suggests that infants living in poorer areas may be exposed to further risk factors (not included in our models) that increase the risk of acute infection and injuries (such as exposure to adverse housing conditions, overcrowding, environmental tobacco smoke) [62–64]. This may result in higher A&E attendance rates and proportionate increases in admission rates, compared to children living in less deprived areas. In addition, research has suggested that access difficulties and dissatisfaction with primary care services, maybe resulting those from in lower SES backgrounds opting to attend hospital rather than in primary care [65–67]. Recent qualitative research has shown young, disadvantaged mothers have complex needs that are not currently addressed by the current systems of care [68].

Nevertheless, infant and socio-economic factors could not explain the substantial variations in A&E attendances and emergency admissions by geographical area. Adding LA random effect accounted for substantial variations in A&E attendances and emergency admissions; highlighting that some unobserved LA-level factors may contribute to some of the variation. These factors could include (but are not limited to) local area level determinants such as primary healthcare availability and accessibility, distance from home to hospital (travel time for parents which would be longer in rural areas compared to urban areas), hospital level admission thresholds, and co-situating general practitioners in A&E departments. In addition, important socio-economic and family related variables were not captured in our data, such as housing characteristics, household income, maternal parity, parental education and occupation [69, 70]. Future research accounting for these factors would yield meaningful understanding of pathways leading to the continued increases in hospital contact. In-depth investigations into local areas performing particularly well or poorly on reducing emergency admissions given their underlying population characteristics would provide insights into cost-effective strategies for reducing need for hospital contacts for families with young infants.

We found rates of A&E attendances and emergency admission for infants continue to increase each year with large regional variations across England; as previously reported [2, 3, 7–12, 17, 18, 71]. London had high A&E attendance rates (876/1000), but the lowest emergency admission rates (232/1000) and conversion proportion (25%). One explanation for this could be due to more Short-Stay Paediatric Assessment Units (SSPAUs; hospital-based facilities where infants with acute illness, injury or other urgent referrals can be assessed, observed and treated with an expectation of discharge in less than 24 hours) being co-located within emergency departments in London compared with the rest of the UK; 35% of SSPAUs in London, compared to approximately 21% across the UK [14]. Further investigation of the route of admissions revealed a substantial proportion of emergency admissions were via direct admissions (primarily via GPs, 92%), where parents did not attend A&E. This was particularly the case outside of London. Conversion rates increased across the study period, potentially suggests an overall system level failure, which may reflect a decrease in funding for community-based postnatal care provision (i.e. in health care visiting and management of neonates in the community following birth discharge) [15, 16], meaning parents may seek care in A&E if they cannot be supported to care for an ill infant at home or elsewhere in the community. Further investigation of detailed LA level factors in future research should lead to better understanding of drivers of geographical variations.

During the first three months of the COVID-19 pandemic, children aged 0–4 years attended A&E departments in England substantially less compared to the same period in 2019 (visits for illnesses 64% lower and visits for injuries 35% lower) [72]. Concerns about parents delaying presentation to emergency departments for high-risk children lead to Royal College of Paediatrics and Child Health (RCPCH) guidelines and alterations in NHS-111 algorithms indicating when A&E visit would be essential (identifying red-flag symptoms) [73–75]. GP telephone and video consultations enabled parents with young children to access healthcare advice remotely [76]. Continuing to improve remote primary care access for parents and tailored preventative guidance for high-risk infants (infants born preterm, with presence of congenital anomaly, and young parent families from low SES background) may help curb pressure on emergency departments. Further studies examining if the trends and patterns in A&E attendances and admissions in infants have changes since the COVID-19 pandemic are needed.

## Conclusion

We identified substantial local area variations in emergency department use among infants, which could not be explained by differences in the underlying prevalence of child, maternal and family risk factors between areas. Future research using more detailed data on individual and family-level risk factors for acute illness and injury in infants (such as on housing and parental occupation), hospital-level factors (such as situating GPs in A&E departments and use of acute assessment unites), and local-area level access and quality of primary care services, would provide insights into potential strategies for ensuring families with young babies can access appropriate urgent care.

## Supplementary Information

**Additional file 1.**

## Data Availability

Source data can be accessed by researchers applying to the Health and Social Care Information Centre for England. Copyright © 2018. Reused with the permission of the Health and Social Care Information Centre.
