# Supplementary material for: Determinants of accident and emergency attendances and emergency admissions in infants: birth cohort study
Source: BMC Health Serv Res. 2022 Jul 21;22:936. doi: 10.1186/s12913-022-08319-1 (PMC9302562; doi:10.1186/s12913-022-08319-1)

Online supplementary material content

[Online supplementary information S1: HES A&E to APC linkage methodology 2](#_Toc76659180)

[Online supplementary Table S2: All cohort socio-demographic and missing data 4](#_Toc76659182)

[Online supplementary Table S3: Incidence risk ratios, coefficients, VPC and model fit statistics from multi-level (mixed effects) negative binomial regression models for outcome A&E attendances……………………………………………………………………………………………...7](#_Toc76659183)

[Online supplementary Table S4: Incidence rate ratios, coefficients, VPC and model fit statistics from multi-level (mixed effects) negative binomial regression models for outcome emergency admissions 9](#_Toc76659184)

[Online supplementary Table S5: Odds ratios, coefficients, VPC and model fit statistics from multi-level (mixed effects) logistic regression models showing Predictors of A&E attendances that become admissions via A&E 11](#_Toc76659185)

[Online supplementary Table S6: Sensitivity analysis of all final fully adjusted models including missing gestational age category 13](#_Toc76659186)

[Online supplementary Figure S7: Probability plots and summary statistics of deviance residuals from final mixed effects negative binomial models for A) A&E attendances and B) Emergency admissions 15](#_Toc76659187)

[Online supplementary Figure S8: Scatter plots (null vs fully adjusted) of predicted local authority effect values (left panel) and ranks (right panel) from mixed effects random intercept negative binomial models for A) A&E attendances and B Emergency admissions 16](#_Toc76659188)

[Online supplementary Figure S9: Probability plots (P-P) plots of random prediction from final mixed effects negative binomial models for A) A&E attendances and B) Emergency admissions 17](#_Toc76659189)

[Online supplementary Figure S10: Scatter plot of deviance residuals for final mixed effects logistic regression model by local authority 18](#_Toc76659190)

[Online supplementary Figure S11: Probability plots (P-P) plots of random prediction from final mixed effects logistic model for conversion probability. 19](#_Toc76659192)

# Online supplementary information S1: HES A&E to APC linkage methodology

We followed the methodology suggested by NHS Digital to link the HES A&E and APC [1]. Briefly, the methodology requires three conditions to be met for data linkage:

1. HESIDs in both datasets are the same
2. The discharge date from A&E and admission date from APC are the same.
3. Episode order in APC is equal to 1

Both A&E and APC datasets were cleaned prior to linkage. For the A&E dataset, each record (row) consisted of information on one A&E attendance. Whereas, in the APC dataset, each record (row) consisted of information on episodes, from which we derived the emergency admissions (inpatient emergency admissions that required the use of a hospital bed). Episodes on each row indicated detailed information on a single episode with a consultant. If the infant then had contact with another consultant, that is recorded as a new row consisting of the second episode. We grouped episodes together to create Inpatient Provider Spells (IPS, periods of continuous care under one NHS provider/hospital). A spell starts when the infant is admitted into hospital (here an admission date is assigned on their record) and ends when the infant is either discharged (indicated by logging of discharge date), transferred to another hospital or if the infant dies. The majority of HES APC records consist of one episode under one NHS provider, but some infants had more complex records consisting of multiple consultant episodes (multi-episode admission). Admissions were classified as an emergency if the first episode within an admission a multi-episode admission was recorded as an emergency episode. Full information on both A&E and APC HES data can be found in relevant data dictionaries [2, 3].

The linkage methodology uses the HESID and a joining date (discharge date from A&E and admission date from APC) for a 1:1 linkage of the A&E and APC dataset; for those who were admitted via an A&E department. These were indicated in the HES A&E datasets using the attendance disposal variable (aeattenddisp code ‘01’) and in the HES APC dataset using the admission method variable (admimeth code ‘21’). The provider code variable (procode) were also required to match. Remaining non-matching records were further matched using different codes within the same variables (see NHS Digital to HES A&E and APC linkage methodology for full details) [1]. Finally, of those that did not match after following full methodology by NHS Digital, we used discharge dates from A&E dataset (plus or minus 1 day) to match further records.

**Linkage quality check**

Matched A&E and APC data records were given a linking score ranging between 1 – 4 to indicate the quality of the link; score of 1 indicated strong link (records met all matching conditions), 2 indicated good link (records did not match on one condition), 3 indicated poor link (record did not met criteria for quality check link 1 or 2) and 4 indicated very poor link (patients that were reported dead on arrival to A&E or died in A&E). For full details of information and criteria see methodology suggested by NHS Digital to link the HES A&E and APC [1].

NHS Digital documentation based on linkage of HES APC and A&E from 2008-09 data illustrated the linkage algorithm and the expected quality check of all linked records were found be a strong link for 85.8%, good link for 13.0%, poor link for 1.2%, and very poor link for 0%. Links that are poor or very poor should be excluded in the final data. Of the records that we linked, the linkage quality check indicated 84.92% as strong link (661,756/779,285), 15.07% as good link (117,462/779,285), and 0.01% as very poor link (67/779,285). We excluded 0.01% of the linked observations that were very poor, which includes infants that were reported either dead on arrival or died while in A&E.

**Non-linking emergency admissions via A&E**

There were a large proportion of APC emergency admissions via A&E that did not link to an A&E attendance, which we expected to have linked (28.97%, 317,493/2,856,397). Further exploration of these observations showed that 82% were from admission method “other means”. We included these non-matching emergency admissions as a part of the analysis if the APC dataset indicated an emergency admission via A&E. This was done to ensure estimates of emergency admissions are not underestimated in comparison to previously reported estimates and because APC data quality is more reliable compared to A&E dataset. We compare our A&E attendance and emergency admissions rates to PHE fingertips official figures to ensure that our estimates follow the same pattern as those previously reported.

References

1. NHS Digital. *HES Accident & Emergency (A&E) to Admitted Patient Care (APC) Linkage Methodology*. 2011 03/09/2019]; Available from: <http://www.macs.hw.ac.uk/~yjc32/project/ref-health%20hospital%20data/AE_to_APC_Linkage_Methodology1.pdf>.

2. NHS Digital. *HES Data Dictionary: Admitted Patient Care*. 2020 08/07/2021]; Available from: <https://digital.nhs.uk/data-and-information/data-tools-and-services/data-services/hospital-episode-statistics/hospital-episode-statistics-data-dictionary>.

3. NHS Digital. *HES Data Dictionary: Accident and Emergency*. 2018 08/07/2021]; Available from: <https://digital.nhs.uk/data-and-information/data-tools-and-services/data-services/hospital-episode-statistics/hospital-episode-statistics-data-dictionary>.

# Online supplementary Table S2: All cohort socio-demographic and missing data

|  |  | All cohort infant/mother | |  | Complete cases  (86%) | | Missing cases (14%) | |  | Crude odds ratios for missing data | | |
| --- | --- | --- | --- | --- | --- | --- | --- | --- | --- | --- | --- | --- |
|  |  | *N* | % |  | Count | % | Count | % |  | OR | 95% CI | |
| Total |  | 4,285,343 | 100 |  | 3,672,700 | 100 | 612,643 | 100 |  |  |  |  |
| Year of birth | 2012/13 | 645,087 | 15.1 |  | 562,914 | 15.3 | 82,173 | 13.4 |  | Ref |  |  |
|  | 2013/14 | 624,247 | 14.6 |  | 533,159 | 14.5 | 91,088 | 14.9 |  | 1.170354 | 1.158539 | 1.18229 |
|  | 2014/15 | 608,897 | 14.2 |  | 524,156 | 14.3 | 84,741 | 13.8 |  | 1.107506 | 1.096149 | 1.11898 |
|  | 2015/16 | 618,499 | 14.4 |  | 527,691 | 14.4 | 90,808 | 14.8 |  | 1.178847 | 1.166934 | 1.190881 |
|  | 2016/17 | 610,067 | 14.2 |  | 518,483 | 14.1 | 91,584 | 14.9 |  | 1.210035 | 1.197821 | 1.222374 |
|  | 2017/18 | 601,546 | 14.0 |  | 522,793 | 14.2 | 78,753 | 12.9 |  | 1.03193 | 1.021178 | 1.042795 |
|  | 2018/19 | 577,000 | 13.5 |  | 483,504 | 13.2 | 93,496 | 15.3 |  | 1.324664 | 1.311314 | 1.338151 |
|  | Missing | 0 | 0.0 |  |  |  |  |  |  |  |  |  |
| Month of birth | January | 353,954 | 8.3 |  | 301,569 | 8.2 | 52,385 | 8.6 |  | Ref |  |  |
|  | February | 320,996 | 7.5 |  | 270,694 | 7.4 | 50,302 | 8.2 |  | 1.06976 | 1.055637 | 1.084072 |
|  | March | 343,462 | 8.0 |  | 287,793 | 7.8 | 55,669 | 9.1 |  | 1.113558 | 1.0992 | 1.128104 |
|  | April | 345,965 | 8.1 |  | 300,070 | 8.2 | 45,895 | 7.5 |  | 0.880486 | 0.868669 | 0.892464 |
|  | May | 368,339 | 8.6 |  | 317,332 | 8.6 | 51,007 | 8.3 |  | 0.925328 | 0.91322 | 0.937596 |
|  | June | 357,243 | 8.3 |  | 309,163 | 8.4 | 48,080 | 7.8 |  | 0.895276 | 0.883397 | 0.907313 |
|  | July | 375,379 | 8.8 |  | 323,821 | 8.8 | 51,558 | 8.4 |  | 0.916581 | 0.904623 | 0.928697 |
|  | August | 369,315 | 8.6 |  | 319,003 | 8.7 | 50,312 | 8.2 |  | 0.907939 | 0.896025 | 0.920011 |
|  | September | 373,170 | 8.7 |  | 321,877 | 8.8 | 51,293 | 8.4 |  | 0.917377 | 0.905394 | 0.929519 |
|  | October | 373,370 | 8.7 |  | 320,238 | 8.7 | 53,132 | 8.7 |  | 0.955131 | 0.942746 | 0.96768 |
|  | November | 351,632 | 8.2 |  | 301,528 | 8.2 | 50,104 | 8.2 |  | 0.956587 | 0.943999 | 0.969343 |
|  | December | 352,518 | 8.2 |  | 299,612 | 8.2 | 52,906 | 8.6 |  | 1.016542 | 1.003317 | 1.029942 |
|  | Missing | 0 | 0.0 |  |  |  |  |  |  |  |  |  |
|  |  | All cohort infant/mother | |  | Complete cases  (86%) | | Missing cases (14%) | |  | Crude odds ratios for missing data | | |
|  |  | *N* | % |  | Count | % | Count | % |  | OR | 95% CI |  |
| **Infant** |  |  |  |  |  |  |  |  |  |  |  |  |
| Infant Sex | Male | 2,198,965 | 51.3 |  | 1,885,887 | 51.3 | 313,078 | 51.2 |  | Ref |  |  |
|  | Female | 2,084,962 | 48.7 |  | 1,786,813 | 48.7 | 298,149 | 48.8 |  | 1.005119 | 0.999689 | 1.010578 |
|  | Missing | 1,416 | 0.0 |  |  |  |  |  |  |  |  |  |
| Gestation at birth | 37+ Term | 3,530,873 | 82.4 |  | 3,460,993 | 94.2 | 69,880 | 91.4 |  | Ref |  |  |
|  | <31: Severe &extreme prematurity | 30,018 | 0.7 |  | 28,834 | 0.8 | 1,184 | 1.5 |  | 2.033736 | 1.917987 | 2.156471 |
|  | 32-33: Moderate prematurity | 25,176 | 0.6 |  | 24,365 | 0.7 | 811 | 1.1 |  | 1.648551 | 1.536546 | 1.768719 |
|  | 34-36: Near Term | 163,098 | 3.8 |  | 158,508 | 4.3 | 4,590 | 6.0 |  | 1.434199 | 1.391414 | 1.478299 |
|  | Missing | 536,178 | 12.5 |  |  |  |  |  |  |  |  |  |
| Congenital anomaly | No | 4,142,450 | 96.7 |  | 3,554,180 | 96.8 | 588,270 | 96.0 |  | Ref |  |  |
|  | Yes | 142,893 | 3.3 |  | 118,520 | 3.2 | 24,373 | 4.0 |  | 1.242453 | 1.225109 | 1.260043 |
|  | Missing | 0 | 0.0 |  |  |  |  |  |  |  |  |  |
| **Geographical area ^a^** |  |  |  |  |  |  |  |  |  |  |  |  |
| IMD Quintile | 0.Most deprived | 1,165,380 | 27.2 |  | 1,020,610 | 27.8 | 144,770 | 26.2 |  | Ref |  |  |
|  | 1 | 951,505 | 22.2 |  | 819,210 | 22.3 | 132,295 | 23.9 |  | 1.13849 | 1.129417 | 1.147636 |
|  | 2 | 790,070 | 18.4 |  | 683,490 | 18.6 | 106,575 | 19.3 |  | 1.099279 | 1.089993 | 1.108644 |
|  | 3 | 682,760 | 15.9 |  | 593,055 | 16.1 | 89,710 | 16.2 |  | 1.066389 | 1.056917 | 1.075945 |
|  | 4.Least deprived | 636,380 | 14.9 |  | 556,335 | 15.1 | 80,045 | 14.5 |  | 1.014332 | 1.005013 | 1.023738 |
|  | Missing data | 59,245 | 1.4 |  |  |  |  |  |  |  |  |  |
|  |  | All cohort infant/mother | |  | Complete cases  (86%) | | Missing cases (14%) | |  | Crude odds ratios for missing data | | |
|  |  | *N* | % |  | Count | % | Count | % |  | OR | 95% CI |  |
| Region | North East | 188,315 | 4.4 |  | 170,285 | 4.6 | 18,030 | 3.4 |  | 0.494038 | 0.486004 | 0.502205 |
|  | North West | 547,900 | 12.8 |  | 497,185 | 13.5 | 50,720 | 9.6 |  | 0.476034 | 0.470921 | 0.481202 |
|  | Yorkshire and Humber | 410,815 | 9.6 |  | 363,925 | 9.9 | 46,830 | 8.9 |  | 0.601243 | 0.594541 | 0.608019 |
|  | East Midlands | 328,305 | 7.7 |  | 299,560 | 8.2 | 28,745 | 5.5 |  | 0.447755 | 0.441794 | 0.453796 |
|  | West Midlands | 454,620 | 10.6 |  | 407,440 | 11.1 | 47,180 | 8.9 |  | 0.540361 | 0.534377 | 0.546412 |
|  | East of England | 456,725 | 10.7 |  | 376,700 | 10.3 | 80,025 | 15.2 |  | 0.991351 | 0.981921 | 1.000871 |
|  | London | 797,420 | 18.6 |  | 656,695 | 17.9 | 140,725 | 26.7 |  | Ref |  |  |
|  | South East | 646,055 | 15.1 |  | 555,520 | 15.1 | 90,535 | 17.2 |  | 0.760516 | 0.75364 | 0.767455 |
|  | South West | 369,750 | 8.6 |  | 345,390 | 9.4 | 24,360 | 4.6 |  | 0.329125 | 0.324481 | 0.333836 |
|  | Missing data | 85,440 | 2.0 |  |  |  |  |  |  |  |  |  |
| **Mother** |  |  |  |  |  |  |  |  |  |  |  |  |
| Age | Under 20 | 149,918 | 3.5 |  | 133,434 | 3.6 | 16,484 | 3.3 |  | 0.798136 | 0.781213 | 0.815425 |
|  | 20 - 29 | 1,839,385 | 42.9 |  | 1,631,168 | 44.4 | 208,217 | 41.8 |  | 0.824704 | 0.81261 | 0.836979 |
|  | 30 - 39 | 2,013,599 | 47.0 |  | 1,762,958 | 48.0 | 250,641 | 50.3 |  | 0.918525 | 0.905156 | 0.932091 |
|  | 40+ | 167,605 | 3.9 |  | 145,140 | 4.0 | 22,465 | 4.5 |  | Ref |  |  |
|  | Missing data | 114,836 | 2.7 |  |  |  |  |  |  |  |  |  |

^a^ NHS Digital Hospital Episode Statistics (HES) disclosure states all HES sub-national data is subject to suppression and rounded to the nearest 5. Therefore, counts for IMD and Region are rounded accordingly (see manuscript ref 30).

# Online supplementary Table S3: Incidence risk ratios, coefficients, VPC and model fit statistics from multi-level (mixed effects) negative binomial regression models for outcome A&E attendances

|  | **Model 1** | |  | **Model 2** | |  | **Model 3** | |
| --- | --- | --- | --- | --- | --- | --- | --- | --- |
|  | Null model with year and month of birth | |  | Model additionally adjusting for infant characteristics sex, gestation at birth, and congenital anomaly | |  | Model additionally adjusting for maternal age and IMD | |
|  | IRR (95% CI) | Coefficient (95% CI) |  | IRR (95% CI) | Coefficient (95% CI) |  | IRR (95% CI) | Coefficient (95% CI) |
| **Fixed effects** |  |  |  |  |  |  |  |  |
| **Year of birth** |  |  |  |  |  |  |  |  |
| 2012/13 (ref) |  |  |  |  |  |  |  |  |
| 2013/14 | 1.28 (1.27 to 1.3) | 0.25 (0.24 to 0.26) |  | 1.2 (1.19 to 1.22) | 0.18 (0.17 to 0.19) |  | 1.20 (1.19 to 1.21) | 0.18 (0.17 to 0.19) |
| 2014/15 | 1.36 (1.34 to 1.38) | 0.31 (0.3 to 0.32) |  | 1.26 (1.25 to 1.28) | 0.23 (0.22 to 0.24) |  | 1.27 (1.26 to 1.28) | 0.24 (0.23 to 0.25) |
| 2015/16 | 1.53 (1.51 to 1.55) | 0.42 (0.41 to 0.44) |  | 1.41 (1.39 to 1.43) | 0.34 (0.33 to 0.36) |  | 1.42 (1.41 to 1.44) | 0.35 (0.34 to 0.36) |
| 2016/17 | 1.57 (1.54 to 1.59) | 0.45 (0.44 to 0.46) |  | 1.45 (1.43 to 1.46) | 0.37 (0.36 to 0.38) |  | 1.46 (1.45 to 1.48) | 0.38 (0.37 to 0.39) |
| 2017/18 | 1.64 (1.62 to 1.67) | 0.5 (0.48 to 0.51) |  | 1.49 (1.47 to 1.51) | 0.4 (0.39 to 0.41) |  | 1.51 (1.49 to 1.53) | 0.41 (0.4 to 0.42) |
| 2018/19 | 2.14 (2.11 to 2.17) | 0.76 (0.75 to 0.78) |  | 2.03 (2 to 2.05) | 0.71 (0.69 to 0.72) |  | 2.06 (2.03 to 2.08) | 0.72 (0.71 to 0.73) |
| **Month of birth** |  |  |  |  |  |  |  |  |
| Q1: January, February, March (ref) |  |  |  |  |  |  |  |  |
| Q2: April, May, June | 0.93 (0.92 to 0.94) | -0.07 (-0.09 to -0.06) |  | 0.92 (0.91 to 0.92) | -0.09 (-0.1 to -0.08) |  | 0.91 (0.91 to 0.92) | -0.09 (-0.1 to -0.08) |
| Q3: July, August, September | 0.96 (0.95 to 0.97) | -0.04 (-0.05 to -0.03) |  | 0.95 (0.94 to 0.95) | -0.05 (-0.06 to -0.05) |  | 0.95 (0.94 to 0.95) | -0.06 (-0.06 to -0.05) |
| Q4: October, November, December | 0.98 (0.97 to 0.99) | -0.02 (-0.03 to -0.01) |  | 0.97 (0.96 to 0.98) | -0.03 (-0.04 to -0.02) |  | 0.97 (0.97 to 0.98) | -0.03 (-0.03 to -0.02) |
| **Sex** |  |  |  |  |  |  |  |  |
| Female (ref) |  |  |  |  |  |  |  |  |
| Male |  |  |  | 1.15 (1.15 to 1.16) | 0.14 (0.14 to 0.15) |  | 1.16 (1.15 to 1.16) | 0.14 (0.14 to 0.15) |
| **Gestation at birth** |  |  |  |  |  |  |  |  |
| 37+ Term (ref) |  |  |  |  |  |  |  |  |
| <32: Severe & extreme prematurity |  |  |  | 1.93 (1.91 to 1.96) | 0.66 (0.65 to 0.67) |  | 1.94 (1.92 to 1.96) | 0.66 (0.65 to 0.68) |
| 32-33: Moderate prematurity |  |  |  | 1.54 (1.51 to 1.56) | 0.43 (0.41 to 0.44) |  | 1.56 (1.54 to 1.58) | 0.44 (0.43 to 0.46) |
| 34-36: Near Term |  |  |  | 1.4 (1.39 to 1.41) | 0.34 (0.33 to 0.35) |  | 1.42 (1.41 to 1.43) | 0.35 (0.35 to 0.36) |
| **Congenital anomaly** |  |  |  |  |  |  |  |  |
| No (ref) |  |  |  |  |  |  |  |  |
| Yes |  |  |  | 2.26 (2.25 to 2.28) | 0.82 (0.81 to 0.82) |  | 2.30 (2.28 to 2.32) | 0.83 (0.83 to 0.84) |
| **Maternal age** |  |  |  |  |  |  |  |  |
| Under 20 |  |  |  |  |  |  | 1.82 (1.81 to 1.84) | 0.6 (0.59 to 0.61) |
| 20 - 29 |  |  |  |  |  |  | 1.31 (1.3 to 1.31) | 0.27 (0.26 to 0.27) |
| 30 - 39 (ref) |  |  |  |  |  |  |  |  |
| 40+ |  |  |  |  |  |  | 0.94 (0.93 to 0.95) | -0.06 (-0.07 to -0.05) |
| **IMD Quintile** |  |  |  |  |  |  |  |  |
| Q1. Most deprived |  |  |  |  |  |  | 1.14 (1.13 to 1.15) | 0.13 (0.12 to 0.14) |
| Q2 |  |  |  |  |  |  | 1.10 (1.09 to 1.11) | 0.09 (0.08 to 0.1) |
| Q3 |  |  |  |  |  |  | 1.05 (1.04 to 1.06) | 0.05 (0.04 to 0.06) |
| Q4 |  |  |  |  |  |  | 1.03 (1.02 to 1.04) | 0.03 (0.02 to 0.04) |
| Q5. Least deprived (ref) |  |  |  |  |  |  |  |  |
|  |  |  |  |  |  |  |  |  |
| **Random effects** |  |  |  |  |  |  |  |  |
| Local authority intercept variance | 0.087 |  |  | 0.078 |  |  | 0.079 |  |
|  |  |  |  |  |  |  |  |  |
| **VPC** |  |  |  |  |  |  |  |  |
| Local authority (level 2) | 0.09 |  |  | 0.11 |  |  | 0.13 |  |
| Individual-level (level 1) | 0.91 |  |  | 0.89 |  |  | 0.87 |  |
|  |  |  |  |  |  |  |  |  |
| **Model fit statistics** |  |  |  |  |  |  |  |  |
|  |  |  |  |  |  |  |  |  |
| BIC | 1,320,148 |  |  | 1,259,346 |  |  | 1,236,159 |  |
|  |  |  |  |  |  |  |  |  |

# Online supplementary Table S4: Incidence rate ratios, coefficients, VPC and model fit statistics from multi-level (mixed effects) negative binomial regression models for outcome emergency admissions

|  | **Model 1** | |  | **Model 2** | |  | **Model 3** | |
| --- | --- | --- | --- | --- | --- | --- | --- | --- |
|  | Null model with year and month of birth | |  | Model additionally adjusting for infant characteristics sex, gestation at birth, and congenital anomaly | |  | Model additionally adjusting for maternal age and IMD | |
|  | IRR (95% CI) | Coefficient (95% CI) |  | IRR (95% CI) | Coefficient (95% CI) |  | IRR (95% CI) | Coefficient (95% CI) |
| **Fixed effects** |  |  |  |  |  |  |  |  |
| **Year of birth** |  |  |  |  |  |  |  |  |
| 2012/13 (ref) |  |  |  |  |  |  |  |  |
| 2013/14 | 1.8 (1.76 to 1.83) | 0.59 (0.57 to 0.61) |  | 1.6 (1.57 to 1.62) | 0.47 (0.45 to 0.48) |  | 1.60 (1.58 to 1.62) | 0.47 (0.46 to 0.49) |
| 2014/15 | 1.89 (1.85 to 1.93) | 0.64 (0.62 to 0.66) |  | 1.66 (1.63 to 1.68) | 0.5 (0.49 to 0.52) |  | 1.67 (1.64 to 1.69) | 0.51 (0.5 to 0.52) |
| 2015/16 | 1.97 (1.93 to 2.01) | 0.68 (0.66 to 0.7) |  | 1.73 (1.71 to 1.76) | 0.55 (0.54 to 0.57) |  | 1.75 (1.72 to 1.77) | 0.56 (0.54 to 0.57) |
| 2016/17 | 1.99 (1.95 to 2.03) | 0.69 (0.67 to 0.71) |  | 1.74 (1.72 to 1.77) | 0.56 (0.54 to 0.57) |  | 1.76 (1.74 to 1.79) | 0.57 (0.55 to 0.58) |
| 2017/18 | 2.06 (2.02 to 2.11) | 0.72 (0.7 to 0.74) |  | 1.78 (1.75 to 1.8) | 0.57 (0.56 to 0.59) |  | 1.8 (1.77 to 1.82) | 0.59 (0.57 to 0.60) |
| 2018/19 | 3.07 (3.01 to 3.13) | 1.12 (1.1 to 1.14) |  | 2.82 (2.77 to 2.86) | 1.04 (1.02 to 1.05) |  | 2.85 (2.81 to 2.90) | 1.05 (1.03 to 1.06) |
| **Month of birth** |  |  |  |  |  |  |  |  |
| Q1: January, February, March (ref) |  |  |  |  |  |  |  |  |
| Q2: April, May, June | 0.9 (0.88 to 0.91) | -0.11 (-0.12 to -0.1) |  | 0.88 (0.87 to 0.89) | -0.13 (-0.14 to -0.12) |  | 0.88 (0.87 to 0.89) | -0.13 (-0.14 to -0.12) |
| Q3: July, August, September | 0.95 (0.94 to 0.96) | -0.05 (-0.07 to -0.04) |  | 0.93 (0.92 to 0.94) | -0.07 (-0.08 to -0.06) |  | 0.93 (0.92 to 0.94) | -0.07 (-0.08 to -0.06) |
| Q4: October, November, December | 0.99 (0.98 to 1) | -0.01 (-0.02 to 0) |  | 0.98 (0.97 to 0.99) | -0.02 (-0.03 to -0.01) |  | 0.98 (0.97 to 0.99) | -0.02 (-0.03 to -0.01) |
| **Sex** |  |  |  |  |  |  |  |  |
| Female (ref) |  |  |  |  |  |  |  |  |
| Male |  |  |  | 1.2 (1.19 to 1.21) | 0.18 (0.17 to 0.19) |  | 1.2 (1.19 to 1.21) | 0.18 (0.18 to 0.19) |
| **Gestation at birth** |  |  |  |  |  |  |  |  |
| 37+ Term (ref) |  |  |  |  |  |  |  |  |
| <32: Severe & extreme prematurity |  |  |  | 2.48 (2.44 to 2.51) | 0.91 (0.89 to 0.92) |  | 2.46 (2.42 to 2.50) | 0.9 (0.88 to 0.92) |
| 32-33: Moderate prematurity |  |  |  | 1.96 (1.92 to 1.99) | 0.67 (0.65 to 0.69) |  | 1.96 (1.92 to 2) | 0.67 (0.65 to 0.69) |
| 34-36: Near Term |  |  |  | 1.7 (1.68 to 1.71) | 0.53 (0.52 to 0.54) |  | 1.7 (1.68 to 1.72) | 0.53 (0.52 to 0.54) |
| **Congenital anomaly** |  |  |  |  |  |  |  |  |
| No (ref) |  |  |  |  |  |  |  |  |
| Yes |  |  |  | 3.40 (3.37 to 3.43) | 1.22 (1.22 to 1.23) |  | 3.42 (3.39 to 3.45) | 1.23 (1.22 to 1.24) |
| **Maternal age** |  |  |  |  |  |  |  |  |
| Under 20 |  |  |  |  |  |  | 1.45 (1.44 to 1.47) | 0.37 (0.36 to 0.39) |
| 20 - 29 |  |  |  |  |  |  | 1.21 (1.2 to 1.22) | 0.19 (0.18 to 0.2) |
| 30 - 39 (ref) |  |  |  |  |  |  |  |  |
| 40+ |  |  |  |  |  |  | 0.94 (0.92 to 0.95) | -0.06 (-0.08 to -0.05) |
| **IMD Quintile** |  |  |  |  |  |  |  |  |
| Q1. Most deprived |  |  |  |  |  |  | 1.09 (1.08 to 1.1) | 0.09 (0.07 to 0.1) |
| Q2 |  |  |  |  |  |  | 1.07 (1.05 to 1.08) | 0.06 (0.05 to 0.08) |
| Q3 |  |  |  |  |  |  | 1.03 (1.02 to 1.04) | 0.03 (0.02 to 0.04) |
| Q4 |  |  |  |  |  |  | 1.02 (1.01 to 1.03) | 0.02 (0.01 to 0.03) |
| Q5. Least deprived (ref) |  |  |  |  |  |  |  |  |
|  |  |  |  |  |  |  |  |  |
| **Random effects** |  |  |  |  |  |  |  |  |
| Local authority intercept variance | 0.159 |  |  | 0.130 |  |  | 0.123 |  |
|  |  |  |  |  |  |  |  |  |
| **VPC** |  |  |  |  |  |  |  |  |
| Local authority (level 2) | 0.09 |  |  | 0.12 |  |  | 0.12 |  |
| Individual-level (level 1) | 0.91 |  |  | 0.88 |  |  | 0.88 |  |
|  |  |  |  |  |  |  |  |  |
| **Model fit statistics** |  |  |  |  |  |  |  |  |
|  |  |  |  |  |  |  |  |  |
| BIC | 1,086,667 |  |  | 997,471 |  |  | 991,385 |  |
|  |  |  |  |  |  |  |  |  |

# **Online supplementary Table S5:** Odds ratios, coefficients, VPC and model fit statistics from multi-level (mixed effects) logistic regression models showing Predictors of A&E attendances that become admissions via A&E

|  | **Model 1** | |  | **Model 2** | |  | **Model 3** | |
| --- | --- | --- | --- | --- | --- | --- | --- | --- |
|  | Null model with year and month of birth | |  | Model additionally adjusting for infant characteristics sex, gestation at birth, and congenital anomaly | |  | Model additionally adjusting for maternal age and IMD | |
|  | OR (95% CI) | Coefficient (95% CI) |  | OR (95% CI) | Coefficient (95% CI) |  | OR (95% CI) | Coefficient (95% CI) |
| **Fixed effects** |  |  |  |  |  |  |  |  |
| **Year of birth** |  |  |  |  |  |  |  |  |
| 2012/13 (ref) |  |  |  |  |  |  |  |  |
| 2013/14 | 1.62 (1.6 to 1.65) | 0.48 (0.47 to 0.5) |  | 1.63 (1.6 to 1.66) | 0.49 (0.47 to 0.51) |  | 1.62 (1.59 to 1.65) | 0.48 (0.47 to 0.5) |
| 2014/15 | 1.58 (1.55 to 1.61) | 0.46 (0.44 to 0.47) |  | 1.58 (1.55 to 1.61) | 0.46 (0.44 to 0.48) |  | 1.57 (1.54 to 1.6) | 0.45 (0.43 to 0.47) |
| 2015/16 | 1.48 (1.46 to 1.51) | 0.39 (0.38 to 0.41) |  | 1.49 (1.46 to 1.51) | 0.4 (0.38 to 0.41) |  | 1.47 (1.45 to 1.5) | 0.39 (0.37 to 0.4) |
| 2016/17 | 1.47 (1.44 to 1.49) | 0.38 (0.37 to 0.4) |  | 1.47 (1.45 to 1.5) | 0.39 (0.37 to 0.4) |  | 1.46 (1.43 to 1.48) | 0.38 (0.36 to 0.39) |
| 2017/18 | 1.51 (1.49 to 1.54) | 0.42 (0.4 to 0.43) |  | 1.52 (1.49 to 1.54) | 0.42 (0.4 to 0.43) |  | 1.5 (1.47 to 1.52) | 0.4 (0.39 to 0.42) |
| 2018/19 | 2.11 (2.08 to 2.15) | 0.75 (0.73 to 0.77) |  | 2.12 (2.08 to 2.16) | 0.75 (0.73 to 0.77) |  | 2.09 (2.05 to 2.13) | 0.74 (0.72 to 0.76) |
| **Month of birth** |  |  |  |  |  |  |  |  |
| Q1: January, February, March (ref) |  |  |  |  |  |  |  |  |
| Q2: April, May, June | 0.92 (0.91 to 0.93) | -0.09 (-0.1 to -0.08) |  | 0.91 (0.9 to 0.92) | -0.09 (-0.1 to -0.08) |  | 0.91 (0.9 to 0.93) | -0.09 (-0.1 to -0.08) |
| Q3: July, August, September | 0.96 (0.95 to 0.97) | -0.04 (-0.05 to -0.03) |  | 0.96 (0.95 to 0.97) | -0.04 (-0.05 to -0.03) |  | 0.96 (0.95 to 0.97) | -0.04 (-0.05 to -0.03) |
| Q4: October, November, December | 1.02 (1.01 to 1.03) | 0.02 (0.01 to 0.03) |  | 1.02 (1.01 to 1.03) | 0.02 (0.01 to 0.03) |  | 1.02 (1.01 to 1.03) | 0.02 (0.01 to 0.03) |
| **Sex** |  |  |  |  |  |  |  |  |
| Female (ref) |  |  |  |  |  |  |  |  |
| Male |  |  |  | 1.05 (1.04 to 1.06) | 0.05 (0.04 to 0.06) |  | 1.05 (1.04 to 1.06) | 0.05 (0.04 to 0.06) |
| **Gestation at birth** |  |  |  |  |  |  |  |  |
| 37+ Term (ref) |  |  |  |  |  |  |  |  |
| <32: Severe & extreme prematurity |  |  |  | 1.84 (1.77 to 1.9) | 0.61 (0.57 to 0.64) |  | 1.84 (1.77 to 1.90) | 0.61 (0.57 to 0.64) |
| 32-33: Moderate prematurity |  |  |  | 1.49 (1.43 to 1.55) | 0.4 (0.36 to 0.44) |  | 1.49 (1.43 to 1.55) | 0.4 (0.36 to 0.44) |
| 34-36: Near Term |  |  |  | 1.47 (1.44 to 1.49) | 0.38 (0.37 to 0.4) |  | 1.47 (1.45 to 1.49) | 0.38 (0.37 to 0.4) |
| **Congenital anomaly** |  |  |  |  |  |  |  |  |
| No (ref) |  |  |  |  |  |  |  |  |
| Yes |  |  |  | 2.17 (2.13 to 2.21) | 0.77 (0.76 to 0.79) |  | 2.17 (2.13 to 2.20) | 0.77 (0.76 to 0.79) |
| **Maternal age** |  |  |  |  |  |  |  |  |
| Under 20 |  |  |  |  |  |  | 0.78 (0.76 to 0.79) | -0.25 (-0.27 to -0.23) |
| 20 - 29 |  |  |  |  |  |  | 0.89 (0.88 to 0.89) | -0.12 (-0.13 to -0.11) |
| 30 - 39 (ref) |  |  |  |  |  |  |  |  |
| 40+ |  |  |  |  |  |  | 1.03 (1.01 to 1.06) | 0.03 (0.01 to 0.05) |
| **IMD Quintile** |  |  |  |  |  |  |  |  |
| Q1. Most deprived |  |  |  |  |  |  | 0.98 (0.97 to 1.00) | -0.02 (-0.03 to 0.00) |
| Q2 |  |  |  |  |  |  | 0.99 (0.98 to 1.01) | -0.00 (-0.02 to 0.01) |
| Q3 |  |  |  |  |  |  | 0.99 (0.98 to 1.01) | -0.01 (-0.02 to 0.01) |
| Q4 |  |  |  |  |  |  | 0.99 (0.99 to 1.01) | -0.00 (-0.01 to 0.01) |
| Q5. Least deprived (ref) |  |  |  |  |  |  |  |  |
|  |  |  |  |  |  |  |  |  |
| **Random effects** |  |  |  |  |  |  |  |  |
| Local authority intercept variance | 0.226898 |  |  | 0.2245288 |  |  | 0.229473 |  |
|  |  |  |  |  |  |  |  |  |
| **VPC (local authority level)** | 0.065 |  |  | 0.064 |  |  | 0.064 |  |
|  |  |  |  |  |  |  |  |  |
| **Model fit statistics** |  |  |  |  |  |  |  |  |
|  |  |  |  |  |  |  |  |  |
| BIC | 1567497 |  |  | 1553462 |  |  | 1552153 |  |
|  |  |  |  |  |  |  |  |  |

# **Online supplementary Table S6:** Sensitivity analysis of all final fully adjusted models including missing gestational age category

|  | A&E attendance final model | |  | Emergency admissions final model | |  | Conversion final model | |
| --- | --- | --- | --- | --- | --- | --- | --- | --- |
|  | IRR (95% CI) | Coefficient (95% CI) |  | IRR (95% CI) | Coefficient (95% CI) |  | OR (95% CI) | Coefficient (95% CI) |
| **Fixed effects** |  |  |  |  |  |  |  |  |
| **Year of birth** |  |  |  |  |  |  |  |  |
| 2012/13 (ref) |  |  |  |  |  |  |  |  |
| 2013/14 | 1.21 (1.2 to 1.22) | 0.19 (0.18 to 0.2) |  | 1.59 (1.57 to 1.61) | 0.46 (0.45 to 0.48) |  | 1.61 (1.58 to 1.64) | 0.48 (0.46 to 0.49) |
| 2014/15 | 1.27 (1.26 to 1.29) | 0.24 (0.23 to 0.25) |  | 1.66 (1.64 to 1.69) | 0.51 (0.5 to 0.52) |  | 1.59 (1.57 to 1.62) | 0.47 (0.45 to 0.48) |
| 2015/16 | 1.42 (1.41 to 1.44) | 0.35 (0.34 to 0.36) |  | 1.75 (1.73 to 1.78) | 0.56 (0.55 to 0.57) |  | 1.50 (1.47 to 1.52) | 0.4 (0.39 to 0.42) |
| 2016/17 | 1.46 (1.45 to 1.48) | 0.38 (0.37 to 0.39) |  | 1.78 (1.76 to 1.81) | 0.58 (0.56 to 0.59) |  | 1.49 (1.47 to 1.52) | 0.4 (0.38 to 0.42) |
| 2017/18 | 1.50 (1.49 to 1.52) | 0.41 (0.4 to 0.42) |  | 1.81 (1.78 to 1.83) | 0.59 (0.58 to 0.6) |  | 1.55 (1.52 to 1.57) | 0.44 (0.42 to 0.45) |
| 2018/19 | 2.07 (2.05 to 2.09) | 0.73 (0.72 to 0.74) |  | 2.85 (2.81 to 2.89) | 1.05 (1.03 to 1.06) |  | 2.13 (2.09 to 2.17) | 0.76 (0.74 to 0.77) |
| **Month of birth** |  |  |  |  |  |  |  |  |
| Q1: January, February, March (ref) |  |  |  |  |  |  |  |  |
| Q2: April, May, June | 0.91 (0.91 to 0.92) | -0.09 (-0.1 to -0.08) |  | 0.88 (0.87 to 0.89) | -0.13 (-0.14 to -0.12) |  | 0.93 (0.92 to 0.94) | -0.08 (-0.09 to -0.07) |
| Q3: July, August, September | 0.95 (0.94 to 0.95) | -0.06 (-0.06 to -0.05) |  | 0.93 (0.92 to 0.94) | -0.07 (-0.08 to -0.06) |  | 0.98 (0.97 to 0.99) | -0.02 (-0.04 to -0.01) |
| Q4: October, November, December | 0.98 (0.97 to 0.98) | -0.02 (-0.03 to -0.02) |  | 0.99 (0.98 to 1) | -0.01 (-0.02 to 0) |  | 1.03 (1.02 to 1.04) | 0.03 (0.02 to 0.04) |
| **Sex** |  |  |  |  |  |  |  |  |
| Female (ref) |  |  |  |  |  |  |  |  |
| Male | 1.16 (1.15 to 1.16) | 0.15 (0.14 to 0.15) |  | 1.20 (1.19 to 1.21) | 0.18 (0.18 to 0.19) |  | 1.05 (1.04 to 1.06) | 0.05 (0.04 to 0.06) |
| **Gestation at birth** |  |  |  |  |  |  |  |  |
| 37+ Term (ref) |  |  |  |  |  |  |  |  |
| <32: Severe & extreme prematurity | 1.93 (1.9 to 1.95) | 0.66 (0.64 to 0.67) |  | 2.44 (2.4 to 2.47) | 0.89 (0.87 to 0.91) |  | 1.83 (1.77 to 1.89) | 0.6 (0.57 to 0.64) |
| 32-33: Moderate prematurity | 1.56 (1.54 to 1.58) | 0.44 (0.43 to 0.46) |  | 1.96 (1.92 to 1.99) | 0.67 (0.65 to 0.69) |  | 1.48 (1.42 to 1.54) | 0.39 (0.35 to 0.43) |
| 34-36: Near Term | 1.42 (1.41 to 1.43) | 0.35 (0.35 to 0.36) |  | 1.70 (1.68 to 1.71) | 0.53 (0.52 to 0.54) |  | 1.46 (1.44 to 1.49) | 0.38 (0.36 to 0.4) |
| Missing data | 1.01 (1.01 to 1.02) | 0.01 (0.01 to 0.02) |  | 1.09 (1.08 to 1.1) | 0.09 (0.08 to 0.1) |  | 1.09 (1.08 to 1.1) | 0.09 (0.07 to 0.1) |
| **Congenital anomaly** |  |  |  |  |  |  |  |  |
| No (ref) |  |  |  |  |  |  |  |  |
| Yes | 2.33 (2.32 to 2.35) | 0.85 (0.84 to 0.85) |  | 3.49 (3.46 to 3.52) | 1.25 (1.24 to 1.26) |  | 2.16 (2.13 to 2.19) | 0.77 (0.75 to 0.79) |
| **Maternal age** |  |  |  |  |  |  |  |  |
| Under 20 | 1.81 (1.8 to 1.83) | 0.6 (0.59 to 0.6) |  | 1.45 (1.43 to 1.47) | 0.37 (0.36 to 0.38) |  | 0.79 (0.78 to 0.8) | -0.24 (-0.25 to -0.22) |
| 20 - 29 | 1.30 (1.3 to 1.31) | 0.26 (0.26 to 0.27) |  | 1.21 (1.2 to 1.22) | 0.19 (0.18 to 0.2) |  | 0.89 (0.88 to 0.9) | -0.12 (-0.12 to -0.11) |
| 30 - 39 (ref) |  |  |  |  |  |  |  |  |
| 40+ | 0.94 (0.93 to 0.95) | -0.06 (-0.07 to -0.05) |  | 0.94 (0.93 to 0.95) | -0.06 (-0.07 to -0.05) |  | 1.03 (1.01 to 1.06) | 0.03 (0.01 to 0.05) |
| **IMD Quintile** |  |  |  |  |  |  |  |  |
| Q1. Most deprived | 1.15 (1.14 to 1.16) | 0.14 (0.13 to 0.15) |  | 1.10 (1.08 to 1.11) | 0.09 (0.08 to 0.1) |  | 0.97 (0.96 to 0.99) | -0.03 (-0.04 to -0.01) |
| Q2 | 1.10 (1.09 to 1.11) | 0.1 (0.09 to 0.11) |  | 1.07 (1.06 to 1.08) | 0.07 (0.06 to 0.08) |  | 0.99 (0.98 to 1.01) | -0.01 (-0.02 to 0.01) |
| Q3 | 1.05 (1.05 to 1.06) | 0.05 (0.04 to 0.06) |  | 1.03 (1.02 to 1.05) | 0.03 (0.02 to 0.04) |  | 1.00 (0.99 to 1.01) | -0.00 (-0.01 to 0.01) |
| Q4 | 1.03 (1.02 to 1.04) | 0.03 (0.02 to 0.04) |  | 1.02 (1.01 to 1.03) | 0.02 (0.01 to 0.03) |  | 0.99 (0.98 to 1.01) | -0.00 (-0.02 to 0.01) |
| Q5. Least deprived (ref) |  |  |  |  |  |  |  |  |
|  |  |  |  |  |  |  |  |  |
| **Random effects** |  |  |  |  |  |  |  |  |
| Local authority intercept variance | 0.077 |  |  | 0.137 |  |  | 0.233 |  |
|  |  |  |  |  |  |  |  |  |
| **VPC**  **(Local authority level)** | 0.12 |  |  | 0.12 |  |  | 0.07 |  |

# **Online supplementary Figure S7:** Probability plots and summary statistics of deviance residuals from final mixed effects negative binomial models for A) A&E attendances and B) Emergency admissions.

1. A&E attendances

3.53% of deviance residuals were outside of the ±1.96 (compared to expected <5%).

Deviance residuals summary statistics:

Range: -3.66 to 10.68

Median: -0.30

Mean: -0.22


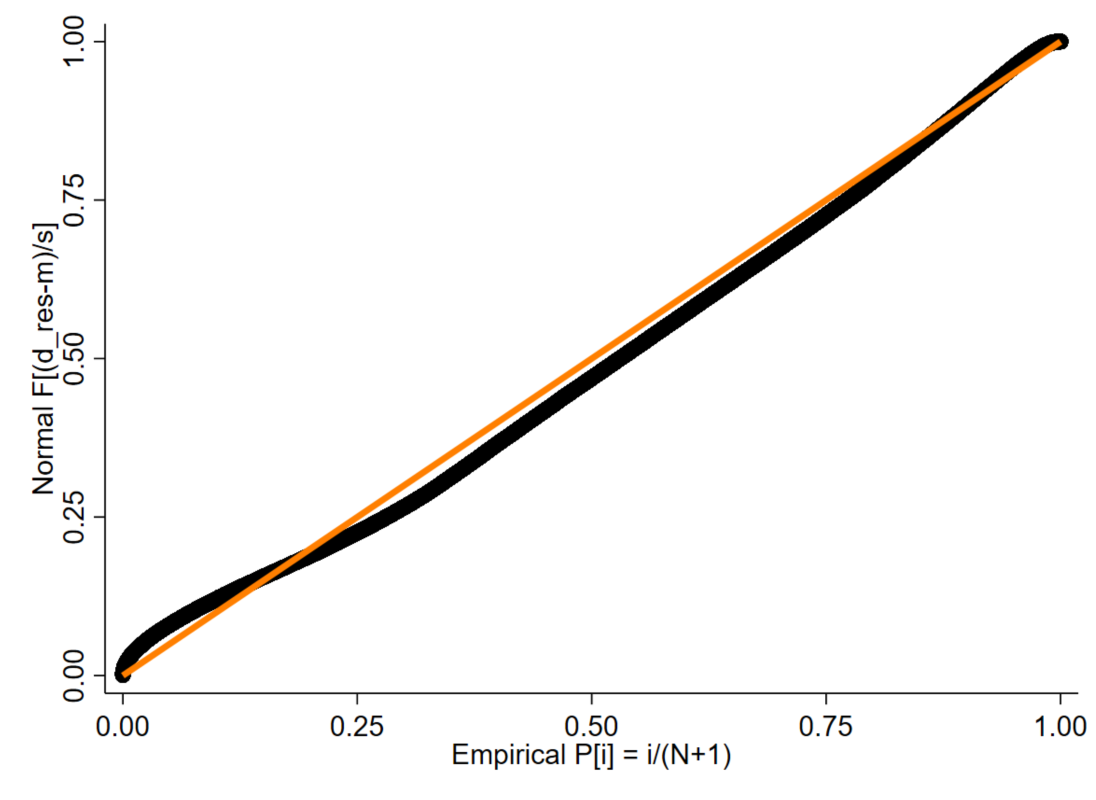


1. Emergency admissions

3.15% of deviance residuals were outside of the ±1.96 (compared to expected <5%).

Deviance residuals summary statistics:

Range: -3.53 to 10.44

Median: -0.43

Mean: -0.24


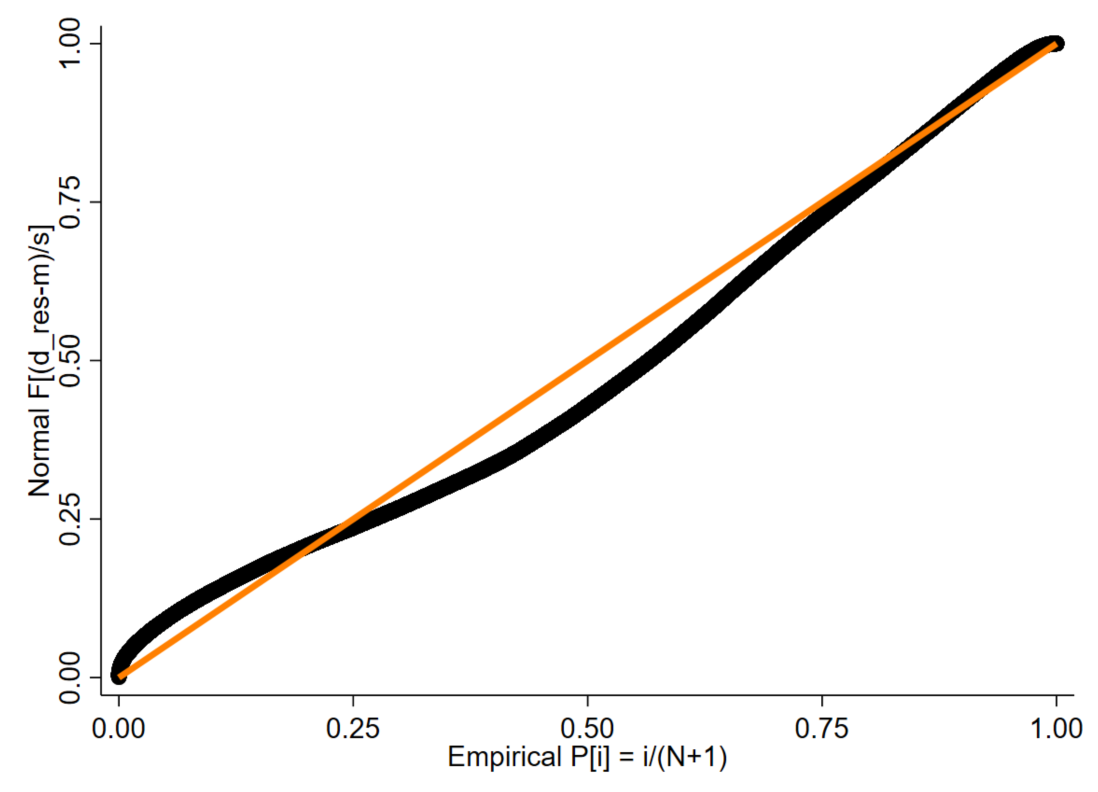


# Online supplementary Figure S8: Scatter plots (null vs fully adjusted) of predicted local authority effect values (left panel) and ranks (right panel) from mixed effects random intercept negative binomial models for A) A&E attendances and B Emergency admissions

**A: A&E attendances**

**B: Emergency admissions**

# **Online supplementary Figure S9:** Probability plots (P-P) plots of random prediction from final mixed effects negative binomial models for A) A&E attendances and B) Emergency admissions

1. A&E attendances


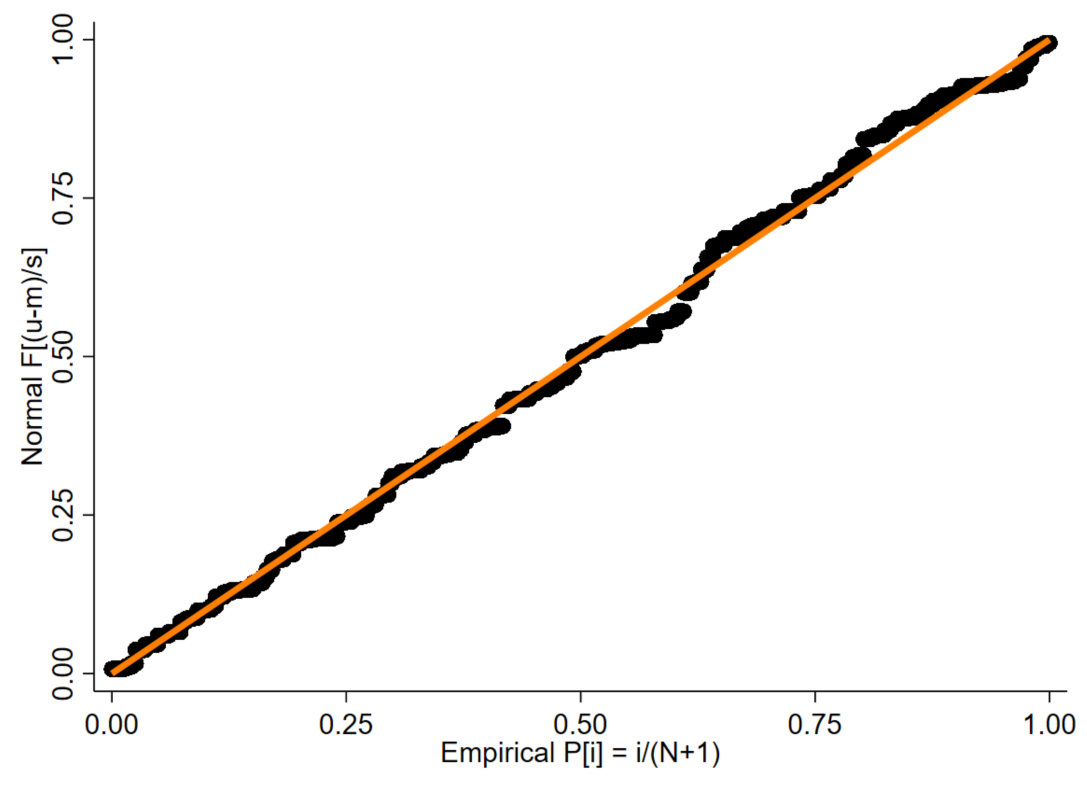


1. Emergency admissions


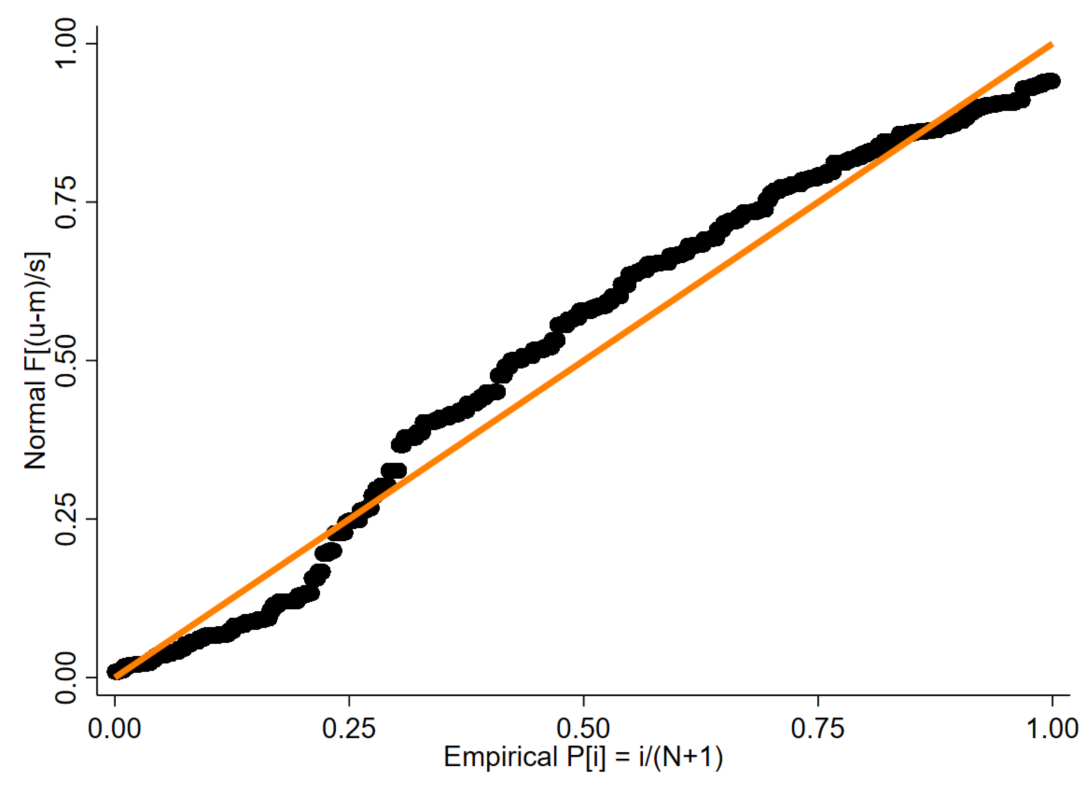


# Online supplementary Figure S10: Scatter plot of deviance residuals for final mixed effects logistic regression model by local authority


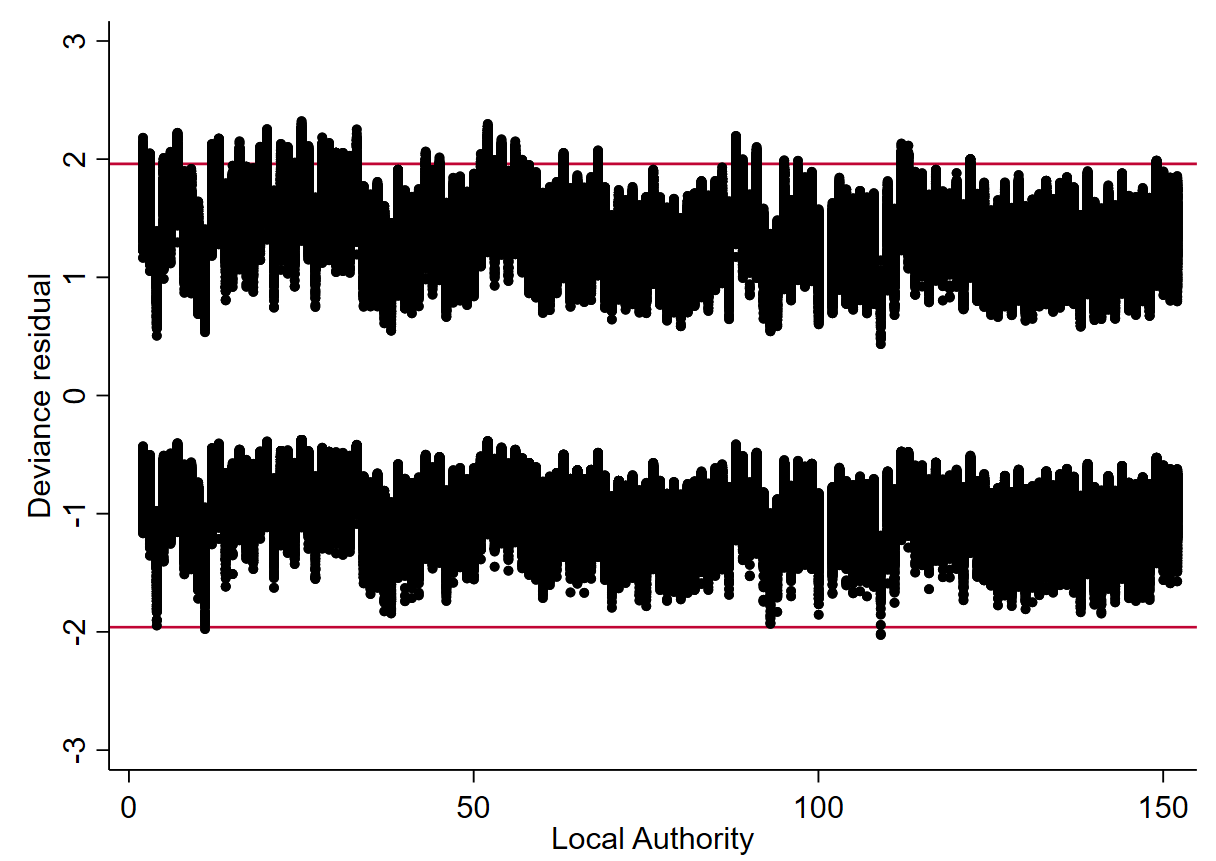


#

0.54% of deviance residuals were outside of the ±1.96 (compared to expected <5%).

Deviance residuals summary statistics:

Range: -2.02 to 2.32

Median: -0.71

Mean: -0.11

# **Online supplementary Figure S11:** Probability plots (P-P) plots of random prediction from final mixed effects logistic model for conversion probability.


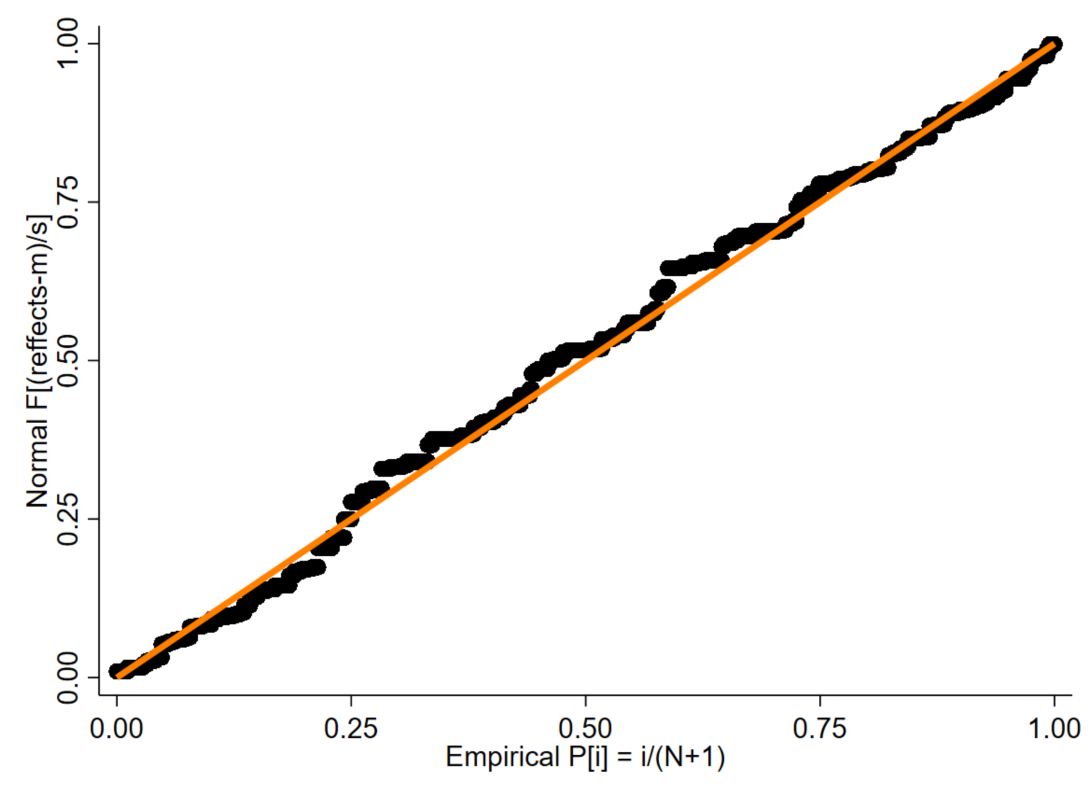

Supplement: Supplementary file 1 — Additional file 1. [file 12913_2022_8319_MOESM1_ESM.docx]
